# A heat transfer model for liquid film boiling on micro-structured surfaces

**DOI:** 10.1093/nsr/nwae090

**Published:** 2024-03-08

**Authors:** Pengkun Li, Qifan Zou, Xiuliang Liu, Ronggui Yang

**Affiliations:** School of Energy and Power Engineering, Huazhong University of Science and Technology, Wuhan 430074, China; School of Energy and Power Engineering, Huazhong University of Science and Technology, Wuhan 430074, China; School of Energy and Power Engineering, Huazhong University of Science and Technology, Wuhan 430074, China; School of Energy and Power Engineering, Huazhong University of Science and Technology, Wuhan 430074, China; State Key Laboratory of Coal Combustion, Huazhong University of Science and Technology, Wuhan 430074, China

**Keywords:** liquid film boiling, heat transfer model, critical heat flux, heat transfer coefficient, thermal management

## Abstract

High heat transfer coefficient (HTC) and critical heat flux (CHF) are achieved in liquid film boiling by coupling vibrant vapor bubbles with a capillary liquid film, which has thus received increased interest for thermal management of high-power electronics. Although some experimental progress has been made, a high-fidelity heat transfer model for liquid film boiling is lacking. This work develops a thermal-hydrodynamic model by considering both evaporation atop the wick and nucleate boiling inside the wick to simultaneously predict the HTC and CHF. Nucleate boiling is modeled with microlayer evaporation theory, where a unified scaling factor is defined to characterize the change of microlayer area with heat flux. The scaling factor *η* is found to be independent of wicking structure and can be determined from a few measurements. This makes our model universal to predict the liquid film boiling heat transfer for various micro-structured surfaces including micropillar, micropowder, and micromesh. This work not only sheds light on understanding fundamental mechanisms of phase-change heat transfer, but also provides a tool for designing micro-structured surfaces in thermal management.

## Teaser

This work develops a high-fidelity heat transfer model for predicting the key performance metrics, heat transfer coefficient and critical heat flux, of liquid film boiling on various micro-structured surfaces.

## INTRODUCTION

Thermal management is becoming increasingly important for advanced electronic and energy systems, such as radars, microprocessors, power inverters, and space systems, where a large amount of heat needs to be dissipated from a limited space and area, and most importantly with a small temperature difference [1–5]. Among various thermal management techniques, liquid-vapor phase-change-based cooling strategies, such as capillary evaporation [6–9] and immersion cooling with pool boiling [10–13], have attracted great attention due to their excellent heat transfer performance. However, conventional cooling methods utilizing liquid-vapor phase-change processes encounter challenges in simultaneously improving the heat transfer coefficient (HTC) and critical heat flux (CHF) on the same wicking structure [4,9,14,15]. Recently, capillary-driven liquid film boiling where vapor bubbles are generated within a wicked liquid film has shown some promise in simultaneously enhancing HTC and CHF [16,17].

Despite the experimental progress on enhancing the heat transfer performance of liquid film boiling on novel micro-structured surfaces [18–22], there still lacks a high-fidelity model that can capture the detailed physical phenomena and predict the CHF and HTC, and guide the design of wicking surfaces. During liquid film boiling, the heat first passes from the heated surface to the solid-fluid matrix of the wick, and then dissipates either by nucleate boiling through vapor bubbles inside the wick or by evaporation through the thin-film region atop the wick [16]. As the heat flux increases, the nucleate boiling becomes dominant, leading to corresponding changes in the heat transfer ratio between nucleate boiling and evaporation atop the wick, as well as the dynamic characteristics of the vapor bubble and liquid film meniscus. Characteristics of such coupling phenomena are further complicated by the variety of wicking structures, which vary both the two-phase capillary delivery and the thermal transport in the solid-fluid matrix [16,23,24]. It is thus challenging but highly desirable to develop a high-fidelity heat transfer model that can account for the thermal-hydraulics and interfacial processes on micro-structured wicking surfaces.

Recently, empirical correlations for CHF of liquid film boiling on the surfaces of micropillar arrays [25] or copper inverse opals [26] have been proposed. It is not known whether these specific fitting formulas based on their own experiments can be used for other structures. Determining the HTC of liquid film boiling under varying heat flux can be much more challenging, as it dynamically varies with the expanded liquid-vapor interfacial area of vapor bubbles coupled with the evaporative meniscus [16,18,20]. Recently, a model was developed for liquid film boiling [27] by assuming a constant liquid-vapor interfacial area, i.e. not varying with heat flux and modeling nucleate boiling inside the wick through pore-scale evaporation. The evaporation atop the wick is neglected. Under these assumptions, the model has good agreement with experimental data after an empirical parameter was adjusted based on the specific wicking structure [27].

In this work, we develop a model to predict both HTC and CHF of liquid film boiling for various micro-structured wicking surfaces. Both evaporation atop the wick and nucleate boiling inside the wick are taken into consideration. Evaporation atop the wick is determined by analyzing the liquid meniscus curvature variation and the thermal resistance network of the thin-film region close to the tri-phase line [6,28]. Nucleate boiling inside the wick is analyzed at pore-scale through the microlayer evaporation borrowed from pool boiling [29–32], with a scaling factor related to heat flux being introduced. Liquid film boiling experiments on micromesh surfaces were conducted along with wick wettability, wicking capability, and structural characterizations, to determine the scaling factor *η.* According to our experiments, the scaling factor *η* is found to be independent of structural parameters, which makes our model universal to predict the liquid film boiling heat transfer for various micro-structured surfaces. Our model predictions are in good agreement with the reported experimental data on various types of micro-structured surfaces, including micromesh, micropowder, and micropillar.

## MODELING AND ANALYSIS

**Figure 1.**
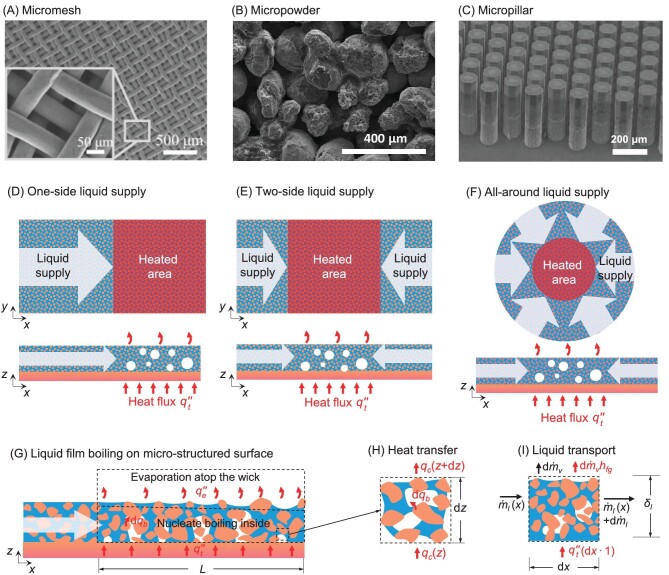
Capillary-driven liquid film boiling on the micro-structured surface. Typical wicking structures include (A) micromesh [16], (B) micropowder [21], and (C) micropillar [25] studied in this work. Common liquid supply methods for liquid film boiling include (D) one-side [16], (E) two-side [33], and (F) all around [21]. (G) Schematic of liquid film boiling heat transfer on a micro-structured surface, where nucleate boiling occurs inside the wicking structure and the evaporation dissipates heat atop the wick. (H) Control volume for heat transfer modeling in the thickness direction (*z*-direction) with a unit width. (I) Control volume for liquid transport along the wicking direction (*x*-direction). Panel (A) is reproduced with permission from ref. [16], Copyright 2018 Elsevier. Panel (B) is reproduced with permission from ref. [21], Copyright 2019 Elsevier. Panel (C) is reproduced with permission from ref. [25], Copyright 2014 Elsevier.

This work aims to develop a unified model for the prediction of HTC and CHF of liquid film boiling on various micro-structured surfaces with different liquid supply methods. Typical microstructures include micromesh, micropowder, and micropillar (Fig. 1A–C), while the liquid can be delivered with one-side [16], two-side [33], and all-around [21] supply methods (Fig. 1D–F). Liquid film boiling with one-side liquid supply on a generalized micro-structured surface is illustrated in Fig. 1G. A set of key structural, thermophysical, and wicking parameters including porosity *ε_w_*, permeability *K_w_*, effective pore radius *r_eff_*, effective thermal conductivity *k_eff_*, and characteristic radius of wick skeleton *r_c_* relating to three typical microstructures, including micromesh, micropowder, and micropillar, are listed in Note S1. Similarly, different liquid supply methods alter liquid delivery boundary conditions as seen in Note S2.

Figure 1G illustrates transport in the *x* to *z* direction, i.e. heat is transferred mainly in the thickness direction (*z*-direction), while the liquid flows along the wicking direction (*x*-direction). On the one hand, heat is absorbed from the heated surface to the liquid film, and transfers inside the liquid film through heat conduction or activates nucleate boiling. On the other hand, the flow of thin liquid film inside the wicking structures depends on both the wicking resistance usually characterized by liquid permeability and capillary pressure, in addition to boiling and vaporization. Here, liquid permeability depends on vapor bubble distribution during the boiling process.

The following assumptions are made to model the physical processes: (1) nucleate boiling occurs uniformly under a constant heat flux across the surface since most wicks are planar-isotropic, perpendicular to the direction of thickness. (2) Heat conduction through the wicked liquid film is one-dimensional along the *z*-direction, since wick thickness along the *z*-direction is generally much smaller than its length along the *x*-direction. (3) The temperature of the vapor within the micropore is uniform along the *z*-direction and equal to the saturated vapor temperature. This is reasonable since the liquid film thickness is typically less than hundreds of micrometers, leading to negligible pressure variation that drives the temperature change [34]. (4) Liquid can be well wicked to maintain a liquid meniscus on top of the wick; the equilibrium liquid meniscus is used to calculate the evaporation atop the wick. (5) For nucleate boiling inside the wick, the microlayer evaporation model can be adopted as has been widely used in pool boiling [29,32,35]. (6) The evaporation atop the wick and nucleate boiling inside the wick can be characterized as heat transfer coefficients *h_e_* and *h_bv_*, respectively.

In the following, we first find the temperature distribution across the wicking structure as a function of the heat transfer coefficient based on the heat balance equation. The heat transfer coefficient *h_e_* of evaporation atop the wick is derived by analyzing the thermal resistance network relating to the curvature of the liquid meniscus. The heat transfer coefficient *h_bv_* of nucleate boiling inside the wick is determined by a pore-scale analysis using the microlayer evaporation theory. The transport of the liquid film inside the wicking structure is modelled using the Brinkman equation. The CHF of boiling can be due to many mechanisms; we consider here the CHF of liquid film boiling to be determined by liquid wicking failure assuming the liquid on the heated surface is completely supplied by surface wicking [26]. By solving the coupled momentum and energy equations, we obtain theoretical expressions for the HTC *h_t_* and the CHF ${{q^{\prime\prime}}_{\!\!\!CHF}}$ of liquid film boiling on various micro-structured surfaces.

### Temperature distribution across the wicking liquid film

Considering a control volume with a unit width inside the wick (Fig. 1H), the heat balance equation can be expressed as *q_c_*(*z*) = *q_c_*(*z* + d*z*) + d*q_b_*, where *q_c_* = ${{q^{\prime\prime}\!\!}_c}$(d*x*⋅1) is the heat conduction rate in the *z*-direction, ${{q^{\prime\prime}\!\!}_c}$ is the conduction heat flux, and *q_b_* is the nucleate boiling heat transfer rate. Given that the conduction heat flux at *z* + d*z* can be expressed as ${{q^{\prime\prime}\!\!}_c}(z + {\mathrm{d}}z) = {{q^{\prime\prime}\!\!}_c}(z) + {\mathrm{d}}{{q^{\prime\prime}\!\!}_c}(z)/{\mathrm{d}}z$, the heat balance equation can then be expressed as:


(1) \begin{eqnarray*}
\frac{{{\mathrm{d}}{{{q^{\prime\prime}}\!\!}_c}(z)}} {{{\mathrm{d}}z}}\left( {{\mathrm{d}}{{x} }\, {\mathrm{d}}z} \right) + {\mathrm{d}} {{q}_b} = 0.
\end{eqnarray*}

We note that an equivalent homogeneous wick is adopted in Fig. 1H for generalization, and this control volume is arbitrarily independent of wicking structure. The conduction heat flux in the *z*-direction is given by:


(2) \begin{eqnarray*}
{q^{\prime\prime}_c}(z) = - {{k}_{eff}}\frac{{{\mathop{\mathrm{d}}\nolimits} {{T}_w}}}{{{\mathrm{d}}z}},
\end{eqnarray*}

where *T_w_* is the temperature of the wicking structure.

Similar to Ref. [27], we assume that heat is dissipated by nucleate boiling with a uniformly volumetric heat transfer coefficient *h_bv_* by averaging the size of bubbles along the wick. The heat transfer rate by nucleate boiling can then be obtained as:


(3) \begin{eqnarray*}
{\mathrm{d}}{{q}_b} = {{h}_{bv}}\!\left( {{{T}_w} - {{T}_v}} \right)\left( {{\mathrm{d}}{{x} }\, {\mathrm{d}}z \cdot 1} \right),
\end{eqnarray*}

where *T_v_* is the vapor temperature. Substituting Eqs. (2) and (3) into Eq. (1), the governing equation of *T_w_* across the wick can be expressed as:


(4) \begin{eqnarray*}
\frac{{{{{\mathrm{d}}}^2}{{T}_w}}}{{{\mathrm{d}}{{z}^2}}}\ = \ \frac{{{{h}_{bv}}}}{{{{k}_{eff}}}}\left( {{{T}_w} - {{T}_v}} \right).
\end{eqnarray*}

Equation (4) is essentially a fin equation, which is a second-order ordinary differential equation (ODE). Two boundary conditions at the bottom (*z* = 0) and top (*z* = *δ_l_*) of the liquid film must be imposed for solving Eq. (4). The heat flux at the heated surface (*z* = 0) is equal to the total heat flux, and this boundary condition can be expressed as [27]:


(5) \begin{eqnarray*}
{{\left. { - {{k}_{eff}}\frac{{{\mathrm{d}}{{T}_w}}}{{{\mathrm{d}}z}}} \right|}_{z = 0}} = {{q^{\prime\prime}\!\!}_t},
\end{eqnarray*}

where ${{q^{\prime\prime}\!\!}_t}$ is the total heat flux. Considering evaporation happens at the top surface of the wick (*z* = *δ_l_*) [16], the boundary condition at the top surface should be written as:


(6) \begin{eqnarray*}
{{\left. {- {{k}_{eff}}\frac{{{\mathrm{d}}{{T}_w}}}{{{\mathrm{d}}z}}} \right|}_{z = {\delta }_l}} = {{q^{\prime\prime}\!\!}_e} = {{h}_e}\!\left( {{{{\left. {{{T}_w}} \right|}}_{z = {{\delta }_l}\ }} - {{T}_v}} \right),
\end{eqnarray*}

where ${{q^{\prime\prime}\!\!}_e}$ and *h_e_* are the heat flux and heat transfer coefficient of the evaporation atop the wick, respectively. *δ_l_* is the thickness of the wicked liquid film. We note that the evaporation atop the wick was neglected in Ref. [27] by writing the boundary condition ${{k}_{eff}}{{ {({\mathrm{d}}{{T}_w}/{\mathrm{d}}z)} |}_{z = {{\delta }_l}}} = 0$.

Integrating Eq. (4) with boundary conditions of Eqs. (5) and (6), the temperature distribution of the wicking structure can be obtained as:


(7) \begin{eqnarray*}
&& {{T}_w}\!\left( z \right) = \frac{{{{{q^{\prime\prime}\!\!}}_t}}}{{m{{k}_{eff}}}}\\
&& \frac{{m{{k}_{eff}}\cosh\! \left[ {m\!\left( {{{\delta }_l} - z} \right)} \right] + {{h}_e}\sinh \left[ {m\!\left( {{{\delta }_l} - z} \right)} \right]}}{{m{{k}_{eff}}\sinh\! \left( {m{{\delta }_l}} \right) + {{h}_e}\cosh\! \left( {m{{\delta }_l}} \right)}}\\
&& +\, {{T}_v},
\end{eqnarray*}

where *m =*  $\sqrt {{{h}_{bv}}/{{k}_{eff}}} $. In the forthcoming sections, *δ_l_* and *h_e_* are determined through analysis of the transport and evaporation of the wicking liquid film, while *h_bv_* is determined by pore-scale analysis of microlayer evaporation. *T_w_*(*z*) can be determined according to Eq. (7) with known *δ_l_, h_e_*, and *h_bv_*, and thus the HTC of liquid film boiling can then be obtained by writing ${{h}_t} = {{q^{\prime\prime}\!\!}_t}/({{ {{{T}_w}} |}_{z = 0}} - {{T}_v})$.

### Transport and evaporation of the wicking liquid film

The liquid film transport inside the wicking structure can be described by the Brinkman equation as [36]:


(8) \begin{eqnarray*}
&& \frac{{{{\partial }^2}{{u}_l}\!\left( {x,z} \right)}}{{\partial {{z}^2}}} - \frac{{{{\varepsilon }_l}}}{{{{K}_{rl}}{{K}_w}}}{{u}_l}\!\left( {x,z} \right) - \frac{{{{\varepsilon }_l}}}{{{{\mu }_l}}}\frac{{{\mathrm{d}}{{P}_l}}}{{{\mathrm{d}}x}} \\
&&- \frac{{{{\varepsilon }_l}}}{{{{\mu }_l}}}{{\rho }_l}g = 0,
\end{eqnarray*}

where *u_l_* is the liquid flow velocity, *ε_l_* is the effective porosity, *μ_l_* is the liquid dynamic viscosity, *K_w_* is wick permeability, *K_rl_* is the relative liquid permeability [37,38], *P_l_* is the local liquid pressure, *ρ_l_* is the liquid density, and *g* is the gravitational acceleration. The boundary conditions for Eq. (8) can be obtained with the non-slip velocity at the substrate and shear-free stress atop the wick as:


(9) \begin{eqnarray*}
\begin{array}{@{}*{1}{c}@{}} {{{{\left. {{{u}_l}\!\left( {x,z} \right)} \right|}}_{z = 0}}{\mathrm{ = }}0,}\\ {{{{\left. {\displaystyle\frac{{\partial {{u}_l}\left( {x,z} \right)}}{{\partial z}}} \right|}}_{z = {{\delta }_l}}} = 0.} \end{array}
\end{eqnarray*}

Equation (8) is a second-order linear ODE, which can be solved with the boundary conditions of Eq. (9) to obtain liquid wicking velocity *u_l_*(*x, z*). Then, the average liquid wicking velocity for arbitrary cross-section at *x* can be expressed as ${{\bar{u}}_l}(x) = \int_{0}^{{{{\delta }_l}}}{{{{u}_l}}}(x,z){\mathrm{d}}z/{{\delta }_l}$ [6], and thus the liquid pressure gradient d*P_l_$/$*d*x* driving the liquid film can be obtained as:


(10) \begin{eqnarray*}
\frac{{{\mathrm{d}}{{P}_l}}}{{{\mathrm{d}}x}} = - \frac{{{{\mu }_l}}}{{{{K}_{rl}}{{K}_w}}}\frac{{{{{\bar{u}}}_l}\!\left( x \right)}}{{\left( {1 - \frac{{\tanh \left( {\\lesssimmbda {{\delta }_l}} \right)}}{{\\lesssimmbda {{\delta }_l}}}} \right)}} - {{\rho }_l}g,
\end{eqnarray*}

where *λ =*  $\sqrt {{{\varepsilon }_l}/{{K}_{rl}}{{K}_w}} $. The relative liquid permeability *K_rl_* in Eq. (10) is computed using the empirical relation as *K_rl_* = *s*^3^ [38], where *s* is the liquid saturation. This liquid saturation *s* is related to nucleate boiling and can be determined by analyzing vapor flow based on the Ergun equation [27] (see Note S3).

A control volume, shown in Fig. 1I, is adopted to depict liquid flows along the *x-*direction due to capillary wicking. The energy balance equation due to phase change can be written as ${{q^{\prime\prime}\!\!}_t}({\mathrm{d}}x \cdot 1) = $*h_fg_*d${\dot{ m}} $*_v_*, where *h_fg_* is the latent heat of vaporization, and $\mathop{\dot{ m}} $*_v_* is the mass flow rate of vapor. Combining the mass conservation as d$\mathop {\dot{m}} $*_l_* + d$\mathop {\dot{m}} $*_v_* = 0, where $\mathop {\dot{m}} $*_l_*(*x*)${\mathrm{ = }}{{\rho }_l}{{\bar{u}}_l}(x){{\delta }_l}(x) \cdot 1$ is the mass flow rate of the liquid, we obtain:


(11) \begin{eqnarray*}
\frac{{{\mathrm{d}}\left[ {{{{\bar{u}}}_l}(x){{\delta }_l}(x)} \right]}}{{{\mathrm{d}}x}} = - \frac{{{{{q^{\prime\prime}}\!\!}_t}}}{{{{\rho }_l}{{h}_{fg}}}}.
\end{eqnarray*}

Since all the liquid evaporated from the surface should enter at *x* = 0, the boundary condition can be set as:


(12) \begin{eqnarray*}
{{\left. {{{{\bar{u}}}_l}\!\left( x \right){{\delta }_l}\!\left( x \right)} \right|}_{x = 0}} = \frac{{{{{q^{\prime\prime}}\!\!}_t}\ L}}{{{{\rho }_l}{{h}_{fg}}}},
\end{eqnarray*}

where *L* is the wicking length as shown in Fig. 1G. Integrating Eq. (11) with the boundary condition of Eq. (12), we obtain the average liquid flow velocity as:


(13) \begin{eqnarray*}
{{\bar{u}}_l}\!\left( x \right) = \frac{{{{{q^{\prime\prime}}\!\!}_t}}}{{{{\rho }_l}{{h}_{fg}}}}\frac{{\left( {L - x} \right)}}{{{{\delta }_l}\!\left( x \right)}}.
\end{eqnarray*}

Substituting Eq. (13) into Eq. (10), the pressure gradient d*P_l_$/$*d*x* can then be obtained as:


(14) \begin{eqnarray*}
\frac{{{\mathrm{d}}{{P}_l}}}{{{\mathrm{d}}x}} = - \frac{{{{\mu }_l}{{{q^{\prime\prime}}\!\!}_t}}}{{{{\rho }_l}{{h}_{fg}}{{K}_w}}}\frac{{\left( {L - x} \right)}}{{{{K}_{rl}}{{\delta }_l}\!\left[ {1 - \frac{{\tanh \left( {\\lesssimmbda {{\delta }_l}} \right)}}{{\\lesssimmbda {{\delta }_l}}}} \right]}} - {{\rho }_l}g.
\end{eqnarray*}

Here, *δ_l_* is related to the curvature of liquid meniscus *H*, which depends on liquid pressure *P_l_* and can be determined by the Young-Laplace equation as *H* = (*P_v_ − P_l_*)/2*σ* [39], with *P_v_* being the vapor pressure and *σ* being the surface tension. Equation (14) is a non-linear first-order ODE for *P_l_*, which can be solved by the four-order Runge-Kutta method with boundary condition ${{ {{{P}_l}} |}_{x = 0}}$= *P_sat_*. The liquid pressure *P_l_* and liquid film thickness *δ_l_* can be obtained accordingly (see Note S4).

With the curvature of the liquid meniscus determined, the heat transfer coefficient *h_e_* of evaporation atop the wick can then be calculated as:


(15) \begin{eqnarray*}
{{h}_e} = {{\left[ {{{A}_{{unit}}}({{R}_{\\smallriptsize\textit{tf}}} + {{R}_{i,e}})} \right]}^{ - 1}}.
\end{eqnarray*}

Here, *A_unit_* is the cross-sectional area of the one-unit cell for the wick, *R_tf_* is thermal conduction resistance through the thin-film region and *R_i,e_* is the thermal resistance for interfacial evaporation [28]. The heat transfer coefficient *h_e_* of evaporation atop the wick is developed based on the saturated vapor environment, which is often seen in liquid film boiling experiments [16,21,23–25]. The detailed calculations of *A_unit_, R_tf_* and *R_i,e_* for different wicking structures can be found in Note S5.

### Heat transfer coefficient of nucleate boiling inside the wick

**Figure 2.**
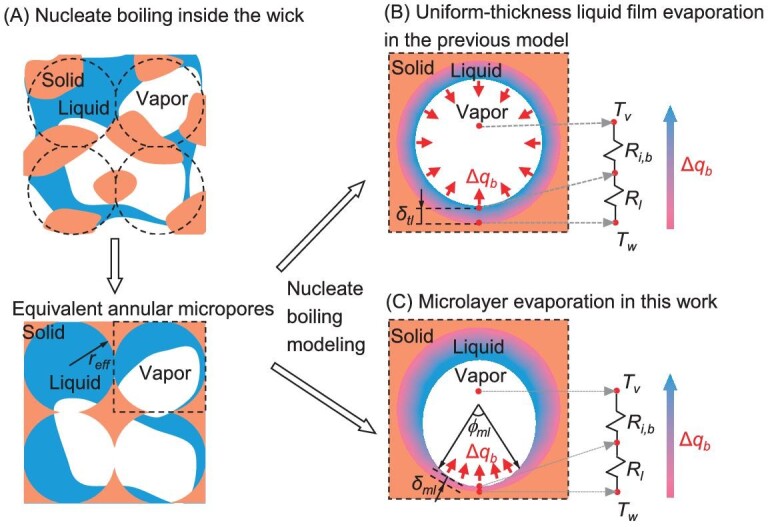
Pore-scale analysis of nucleate boiling inside the wicking structure. (A) Nucleate boiling inside the wick micropores, which can be equivalent to annular interconnected micropores [27]. (B) Nucleate boiling in a micropore unit is modeled as evaporation with uniform thickness of the thin liquid film in the previous model [27]. (C) Nucleate boiling in a micropore unit is modeled as microlayer evaporation at the microlayer region with an average thickness of *δ_ml_* in this work.

For capillary-driven liquid film boiling, there are two typical types of microstructure: porous structures with interconnected micropores and micro-pillared surface. For the former, we use a representative micropore unit within the wick to analyze the nucleate boiling inside the wick. This micropore unit can be conceptualized as an annular pore with an effective pore radius of *r_eff_*, as shown in Fig. 2A. This assumption is reasonable when the effective structural characteristics, including effective pore radius and porosity, are used to ensure the same capillary force and permeability for liquid wicking and bubble escaping. For the micro-pillared surface, we use a micropillar unit to analyze the nucleate boiling inside the wick. Since the analysis of both types of structures is similar, we will present here only the model derivation for the porous structures with interconnected micropores. The derivation for the micropillar can be found in Note S6. The micropore unit approximation for porous structures was also used in earlier studies [27] to analyze the nucleate boiling, where the nucleate boiling was assumed to be thin-film evaporation with a uniform liquid film thickness *δ_tl_* independent of heat flux (Fig. 2B). In Ref. [27], a constant liquid-vapor evaporation area is assumed, resulting in a constant heat transfer coefficient *h_bv_* as:


(16) \begin{eqnarray*}
{{h}_{bv}} = \frac{{2{{\varepsilon }_w}{{k}_l}}}{{r_{eff}^2\left( {\ln\! \left( {\frac{{{{r}_{eff}}}}{{{{r}_{eff}} - {{\delta }_{tl}}}}} \right) + \frac{{{{k}_l}}}{{{{h}_i}\left( {{{r}_{eff}} - {{\delta }_{tl}}} \right)}}} \right)}}.
\end{eqnarray*}

Indeed, under higher heat flux, the effective evaporation area due to nucleate boiling should change with the heat flux due to the different fractions of bubbles activated.

Similar to microlayer evaporation analysis for pool boiling widely used in the literature [29,32,35], we adopt microlayer evaporation to model nucleate boiling inside the wicking structures at various heat fluxes. As shown in Fig. 2C, the heat within the micropore mainly transfers through a microlayer region characterized by a thickness of *δ_ml_*. For saturated water, the microlayer has a thickness *δ_ml_* that typically ranges between 1 and 10 μm [29], which is similar to the thin-film region for evaporation atop the wick with a thickness <0.15*r_c_* [7], where *r_c_* is the characteristic radius of the solid skeleton of the wick (see Note S1). An averaged thickness *δ_ml_* of microlayer region is used here to estimate *h_bv_* by setting *δ_ml_* = 0.15*r_c_*/2 in our calculations.

The nucleate boiling heat transfer can be considered as heat transfers through a microlayer conduction resistance *R_l_* from the wall to the liquid-vapor interface, then evaporates with an interfacial resistance *R_i,b_*, given by:


(17) \begin{eqnarray*}
{{R}_l} = \int\limits_{{{{r}_{eff}} - {{\delta }_{ml}}}}^{{{{r}_{eff}}}}{{\frac{{{\mathrm{d}}r}}{{{{k}_l}{{\phi }_{ml}}r \cdot 1}}}} = \frac{1}{{{{k}_l}{{\phi }_{ml}}}}\ \ln\! \left( {\frac{{{{r}_{eff}}}}{{{{r}_{eff}} - {{\delta }_{ml}}}}} \right),
\end{eqnarray*}

(18) \begin{eqnarray*}
{{R}_{i,b}} = \frac{1}{{{{h}_i}{{A}_{ml}}}} = \frac{1}{{{{h}_i}\!\left( {{{r}_{eff}} - {{\delta }_{ml}}} \right){{\phi }_{ml}} \cdot 1}},
\end{eqnarray*}

where *ϕ_ml_* is the center angle corresponding to the microlayer region in the annular micropore, *A_ml_* is the liquid-vapor interfacial area of the microlayer region (or microlayer area), *k_l_* is the thermal conductivity of the liquid, and *h_i_* is the heat transfer coefficient for the liquid-vapor interface [40]. Thus, *h_i_* can be obtained from the Schrage equation [41], with an accommodation coefficient of 0.04 chosen for water [28,40]. The total heat dissipated by microlayer evaporation Δ*q_b_* within the micropore can be expressed as Δ*q_b_* = (*T_w_ − T_v_*)/(*R_l_* + *R_i,b_*).

Assuming the microlayer evaporation dissipates heat with an equivalent volume-averaged heat transfer coefficient *h_bv_*, Δ*q_b_* can also be expressed as Δ*q_b_* = *h_bv_V_up_*(*T_w_* − *T_v_*), where *V_up_* = *V_pore_*/*ε_w_* is the total volume of unit, *V_pore_* = *πr_eff_*^2^⋅1 = *r_eff_A_total_*/2 is the volume of the micropore unit, and *A_total_* is the surface area. The volumetric heat transfer coefficient of nucleate boiling *h_bv_* can then be obtained as:


(19) \begin{eqnarray*}
&&{{h}_{bv}} =\\
&& \frac{{2{{\varepsilon }_w}{{k}_l}}}{{{{r}_{eff}}\!\left( {{{r}_{eff}} - {{\delta }_{ml}}} \right)\left( {\ln\! \left( {\frac{{{{r}_{eff}}}}{{{{r}_{eff}} - {{\delta }_{ml}}}}} \right) + \frac{{{{k}_l}}}{{{{h}_i}\left( {{{r}_{eff}} - {{\delta }_{ml}}} \right)}}} \right)}}\\
&&\times \, \frac{{{{A}_{ml}}}}{{{{A}_{{total}}}}}.
\end{eqnarray*}

Combining the analysis for the micro-pillared surface (see Note S6), a general form of *h_bv_* can be then obtained as:


(20) \begin{eqnarray*}
&& {{h}_{bv}}= \\
&& \frac{{2{{\varepsilon }_w}{{k}_l}}}{{{{r}_{eff}}\left( {{{r}_{eff}} \!+\! \chi {{\delta }_{ml}}} \right)\left( {\frac{1}{\chi }\ln \left( {1 \!+\! \frac{{\chi {{\delta }_{ml}}}}{{{{r}_{eff}}}}} \right) \!+\! \frac{{{{k}_l}}}{{{{h}_i}\left( {{{r}_{eff}} \!+\! \chi {{\delta }_{ml}}} \right)}}} \right)}}\\
&&\times \, \frac{{{{A}_{ml}}}}{{{{A}_{{total}}}}},
\end{eqnarray*}

where *χ* is a structural factor: *χ* = −1 is used for the porous medium such as micropowder and micromesh, while *χ* = *ε_w_*/(1 − *ε_w_*) is used for micro-pillared surface. The term *A_ml_$/$A_total_* represents the area fraction of the microlayer region to the total heated surface. As shown in pool boiling, the microlayer area increases with the heat flux [29,31,32,42], and an approximately linear function between *A_ml_$/$A_total_* and heat flux was recently proposed [32], i.e. *A_ml_$/$A_total_*  $\propto\ {{q^{\prime\prime}\!\!}_t}$. Since the microlayer evaporation in pool boiling and liquid film boiling are similar, this linear function between the microlayer area fraction (*A_ml_$/$A_total_*) and the total heat flux is also adopted in this work, i.e. *A_ml_$/$A_total_* = $\eta {{q^{\prime\prime}\!\!}_t}$ by introducing *η* as a scaling factor. Equation (20) can then be reformulated as:


(21) \begin{eqnarray*}
&& {{h}_{bv}}= \\
&& \frac{{2{{\varepsilon }_w}{{k}_l}}}{{{{r}_{eff}}\!\left( {{{r}_{eff}} + \chi {{\delta }_{ml}}} \right)\left( {\frac{1}{\chi }\ln\! \left( {1 + \frac{{\chi {{\delta }_{ml}}}}{{{{r}_{eff}}}}} \right) + \frac{{{{k}_l}}}{{{{h}_i}\left( {{{r}_{eff}} + \chi {{\delta }_{ml}}} \right)}}} \right)}}\eta {{q^{\prime\prime}\!\!}_t}.\\
\end{eqnarray*}

Equation (21) shows that larger heat flux ${{q^{\prime\prime}\!\!}_t}$, higher porosity *ε_w_*, and smaller effective pore radius *r_eff_* can lead to higher heat transfer coefficient of nucleate boiling *h_bv_*. In this work, the empirical parameter *η* is obtained by fitting the model predictions to the experimental results of liquid film boiling, with the details provided in Section 3.

### HTC and CHF of liquid film boiling

With the obtained *h_e_* and *h_bv_*, the temperature distribution of the wicking liquid film along the *z*-direction can be calculated from Eq. (7). Specifically, the wall temperature ${{ {{{T}_w}} |}_{z = 0}}$ can then be expressed as:


(22) \begin{eqnarray*}
{{\left. {{{T}_w}} \right|}_{z = 0}} = \frac{{{{{q^{\prime\prime}}\!\!}_t}}}{{m{{k}_{eff}}}}\ \frac{{m{{k}_{eff}} + {{h}_e}\tanh\! \left( {m{{\delta }_l}} \right)}}{{{{h}_e} + m{{k}_{eff}}\tanh\! \left( {m{{\delta }_l}} \right)}} + {{T}_v}.
\end{eqnarray*}

Since *h_e_* varies with wicking distance *x*, an average wall temperature is adopted as:


(23) \begin{eqnarray*}
&&{{\left. {{{{\bar{T}}}_w}} \right|}_{z = 0}} = \frac{{\int\nolimits_{0}^{L}{{{{{\left. {{{T}_w}} \right|}}_{z = 0}}{\mathrm{d}}x}}}}{L}\\
&& = \frac{{{{{q^{\prime\prime}}\!\!}_t}}}{{m{{k}_{eff}}L}} \int\nolimits_{0}^{L}{{\frac{{m{{k}_{eff}} + {{h}_e}\tanh\! \left( {m{{\delta }_l}} \right)}}{{{{h}_e} + m{{k}_{eff}}\tanh\! \left( {m{{\delta }_l}} \right)}}{\mathrm{d}}x}} \!+\! {{T}_v}.\\
\end{eqnarray*}

The HTC of liquid film boiling can then be calculated as ${{h}_t} = {{q^{\prime\prime}\!\!}_t}/({{ {{{{\bar{T}}}_w}} |}_{z = 0}} - {{T}_v})$, with


(24) \begin{eqnarray*}
{{h}_t} = \frac{{m{{k}_{eff}}L}}{{\int\limits_{0}^{L}{{\frac{{m{{k}_{eff}} + {{h}_e}\tanh \left( {m{{\delta }_l}} \right)}}{{{{h}_e} + m{{k}_{eff}}\tanh \left( {m{{\delta }_l}} \right)}}{\mathrm{d}}x}}}}.
\end{eqnarray*}

We note that the previous model [27] simplified the liquid film thickness *δ_l_* as the wick thickness *δ_w_* (i.e. *δ_l_* ≈ *δ_w_*) and ignored evaporation atop the wick, i.e. *h_e_* = 0. By applying these simplifications to Eq. (24), we have *h_t_* = *mk_eff_*tanh(*mδ_w_*), which is the same result as derived in Ref. [27]. When no bubbles nucleate (i.e. *h_bv_* = 0), Eq. (24) reduces to *h_t_* = 1/(1/*h_e_*+*δ_w_*/*k_eff_*), which is consistent with the HTC correlation developed for capillary evaporation [6]. By such limit analysis, we can see that our model is more general and can be reduced to those in Ref. [27] and for capillary evaporation.

The CHF of liquid film boiling occurs when the liquid pressure drop is equal to the maximum capillary pressure *P_c_*,_max_ in the wick [6], i.e.$- \int_{0}^{L}{{{{{({\mathrm{d}}{{P}_l}/{\mathrm{d}}x)}}^{}}}}{\mathrm{d}}x = {{P}_{c,\max }}$. By combining this equation with Eq. (14), the relation of CHF for liquid film boiling can be obtained as:


(25) \begin{eqnarray*}
{{q^{\prime\prime}\!\!}_{CHF}} = \displaystyle\frac{1}{{{{\mu }_l}}}\displaystyle\frac{{{{\rho }_l}{{h}_{fg}}{{K}_w}\!\left( {{{P}_{c,\max }} - {{\rho }_l}gL} \right)}}{{\mathop \smallint \limits_0^L \displaystyle\frac{{\left( {L - x} \right)}}{{{{K}_{rl}}{{\delta }_l}\!\left[ {1 - \tanh\! \left( {\\lesssimmbda {{\delta }_l}} \right)/\left( {\\lesssimmbda {{\delta }_l}} \right)} \right]}}{\mathrm{d}}x}}.
\end{eqnarray*}

From Eq. (25), it is evident that increasing capillary force *P_c_*_, max_, increasing wick permeability *K_w_*, and augmenting relative liquid permeability *K_rl_* can enhance the CHF. Since *K_rl_* is an exponential function of the liquid saturation *s* [27,37], increasing the liquid saturation by promoting vapor escape can also enhance the CHF. Neglecting substrate friction (i.e. neglecting no-slip boundary condition and assuming uniform velocity along the *z*-direction at each *x* with $\partial {{u}_l}/\partial z = 0$), assuming uniform vapor distribution across the liquid film (i.e. the relative liquid permeability *K_rl_* to be constant), Eq. (25) reduces to ${{q^{\prime\prime}\!\!}_{CHF}} = 2{{P}_{c,\max }}{{\rho }_l}{{h}_{fg}}{{\delta }_w}{{K}_{rl}}{{K}_w}/{{\mu }_l}{{L}^2}$, which is similar to the CHF correlation for liquid film boiling proposed by Zhang *et al*. [26]. When simplifying liquid film thickness to become the wick thickness (*δ_l_* ≈ *δ_w_*), neglecting gravitational effect (i.e. *g* = 0), and assuming the *K_rl_* equals 1 (indicating that no bubbles exist inside the wick), Eq. (25) can be reduced to ${{q^{\prime\prime}\!\!}_{CHF}} = 2{{P}_{c,\max }}{{\rho }_l}{{h}_{fg}}{{\delta }_w}{{K}_w}[ {1 - \tanh (\\lesssimmbda {{\delta }_w})/\\lesssimmbda {{\delta }_w}} ]/{{\mu }_l}{{L}^2}$, which is consistent with the CHF correlation to predict the dry-out heat flux of capillary evaporation [43]. In Note S7, we also compare the heat transfer performance between liquid film boiling and capillary evaporation using our model.

## EXPERIMENTAL DETERMINATION OF THE SCALING FACTOR ***η***

**Figure 3.**
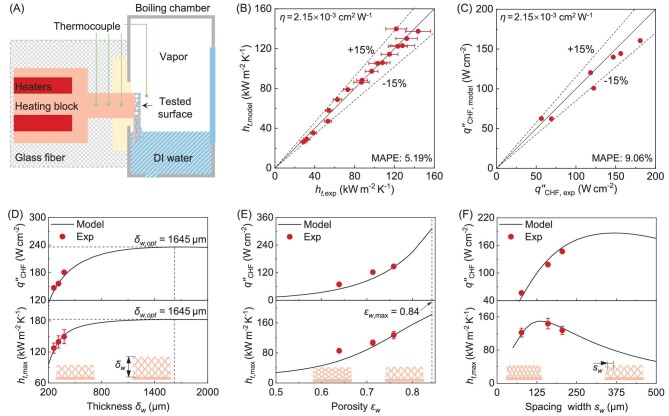
Determination of effective microlayer evaporation factor *η* by experimental results. (A) Schematic of the custom-made experimental setup for liquid film boiling measurement. (B) Determination of *η* = 2.15 × 10^−3^ cm^2^ W^−1^ with our experimental data on copper micromesh samples s1 and s2. (C) Comparison of experimental CHF and model-predicted CHF with *η* = 2.15 × 10^−3^ cm^2^ W^−1^. The CHF and the maximum HTC as the function of (D) thickness *δ_w_*, (E) porosity *ε_w_*, and (F) spacing width *s_w_*. The red circles in (D–F) represent the experimental data and the black solid lines are the modeling results with *η* = 2.15 × 10^−3^ cm^2^ W^−1^.

The scaling factor *η* in Eq. (21) characterizes the relationship between microlayer area fraction and heat flux for liquid film boiling, which is difficult to determine theoretically due to a lack of understanding of the bubble dynamics of liquid film boiling. Instead, we theoretically analyze the *η* value of pool boiling in Note S8. Considering the difference in the transport processes between the pool boiling and liquid film boiling, we here obtain the *η* value by fitting our model predictions with our experimental data on sintered multi-layer micromesh surfaces. In this work, the physical properties of the micromesh, such as porosity, permeability, and wettability, which are not fully presented in the existing literature, are characterized (see Note S9). Liquid film boiling experiments are conducted using a custom-made experimental system [16,28], which provides a saturated vapor environment for liquid film boiling (Fig. 3A). The tested sample is mounted vertically to the heating block with a 10 × 10 mm^2^ heating area and immersed in degassed deionized water with one-side liquid supply (see Note S10), consistent with our previous works [16,20,28]. The heat flux ${{q^{\prime\prime}\!\!}_t}$ and surface temperature ${{ {{{T}_w}} |}_{z = 0}}$ are obtained from the one-dimensional temperature distribution in the heating block. The measured HTC is calculated as ${{h}_t} = {{q^{\prime\prime}\!\!}_t}/({{ {{{T}_w}} |}_{z = 0}} - {{T}_v})$.

The experimental data on micromesh samples s1 and s2, which have similar wire diameter (∼50 μm) and thickness (∼265 μm) but different spacing width (77 and 160 μm, respectively), is used to obtain the *η* value. The model prediction of HTC is obtained from Eq. (24) based on the structure properties as shown in Table S5. To maintain consistency with the experiment, we include an extra heat conduction resistance of the substrate in the model-predicted HTC calculation, as 1/(1/*h_t_* + *δ_sub_*/*k_sub_*), where *h_t_* is calculated by Eq. (24), *δ_sub_* is the thickness of the substrate in the experiment, and *k_sub_* is the thermal conductivity of the substrate. This calculation method is also used in the following sections when comparing model-predicted HTC with experimental data. The factor *η* in Eq. (21) is determined to be *η* = 2.15 × 10^−3^ cm^2^ W^−1^ by fitting the model prediction of HTC with our experimental data on such uniform micromesh samples, with a mean absolute percentage error (MAPE) of 5.19% (Fig. 3B). Such a unified value of *η* could then be used to estimate the microlayer area fraction of the uniform wicking surface according to *A_ml_$/$A_total_* = *η*${{q^{\prime\prime}\!\!}_t}$. Figure 3C shows the model-predicted CHF for different uniform micromesh samples, with thickness ranging from 260 to 377 μm, porosity ranging from 0.57 to 0.76, and spacing width ranging from 77 to 205 μm. With the same value of *η* = 2.15 × 10^−3^ cm^2^ W^−1^, our CHF model agrees well with experimental data, with ±15% accuracy (Fig. 3C).

Figure 3D–F show the effects of thickness *δ_w_*, porosity *ε_w_*, and spacing width *s_w_* on CHF and maximum HTC (HTC at CHF). The CHF is enhanced with increased micromesh thickness (Fig. 3D), due to enhanced wick permeability and decreased resistance of the liquid flow [6]. However, increasing the wick thickness could also increase the vapor escaping resistance and sightly decrease the CHF. Although increasing the porosity of the micromesh also increases the liquid wicking velocity, there exists an upper limit for the wick porosity for the micromesh with the same wire diameter and the same spacing width (Fig. 3E). Increasing the spacing width enhances wick permeability, however, the maximum capillary force is reduced due to the enlarged effective pore radius [44], thereby yielding an optimal spacing width *s_w_* for CHF (Fig. 3F). The maximum HTC is mainly controlled by the maximum microlayer area *A_ml_*,_max_ for nucleate boiling, calculated as *A_ml_*,_max_ = 2.15 × 10^−3^${{q^{\prime\prime}\!\!}_t}$*A_total_*, where *A_total_* = $N \times (2\pi {{r}_{eff}} \cdot 1) = ({{V}_{{total}}}{{\varepsilon }_w}/\pi r_{eff}^2) \times (2\pi {{r}_{eff}} \cdot 1) = 2{{V}_{{total}}}{{\varepsilon }_w}/{{r}_{eff}}$ is the total area of the micropore with *N* being the number of micropores and *V_total_* being the total wick volume. As shown in Fig. 3D and E, the maximum HTC is increased with larger thickness and increased porosity since the maximum microlayer area for evaporation is expanded with an increased CHF ${{q^{\prime\prime}\!\!}_{CHF}}$ and the expanded total micropore area *A_total_.* The effect of spacing width *s_w_* on the maximum HTC is multi-faceted. Though increasing *s_w_* augments porosity to expand the total micropore area *A_total_*, it simultaneously increases the effective pore radius *r_eff_* and may reduce the total micropore area *A_total_.* Besides, ${{q^{\prime\prime}\!\!}_{CHF}}$ initially increases with spacing width and subsequently decreases, which also accordingly changes *A_ml_*,_max_. Consequently, the competition results in an optimal *s_w_* to maximize effective *h_bv_* for nucleate boiling and HTC (Fig. 3F). From Fig. 3D–F, we can clearly see that our model with the same value of *η* = 2.15 × 10^−3^ cm^2^ W^−1^ can predict both maximum HTC and CHF with high fidelity for micromesh with different structural parameters. Although changing the wick geometry changes both the total surface area *A_total_* and the effective microlayer region area *A_ml_*, the area fraction *A_ml_$/$A_total_* is effectively estimated by the scaling factor *η* in our model.

## RESULTS AND DISCUSSION

### Model validation

**Figure 4.**
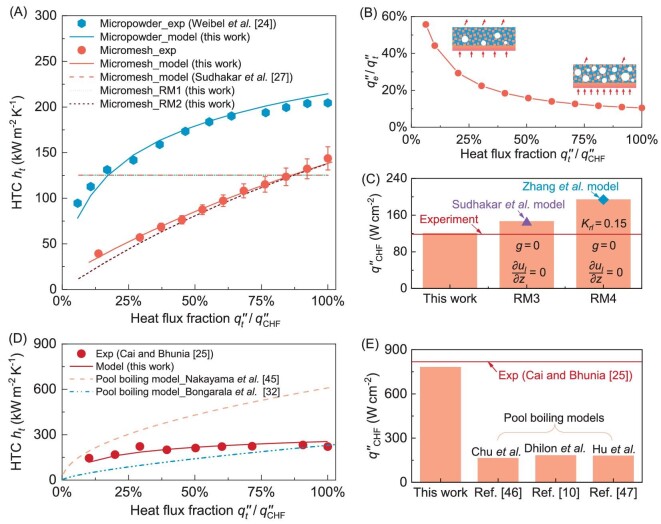
Model validation with experimental data and simplifications. (A) Comparison of model-predicted HTC with the experimental data and prediction from other models [27]. The experimental data on micromesh sample s2 (*d_w_* = 50 μm, *s_w_* = 160 μm, *δ_w_* = 264 μm and *ε_w_* = 0.71) are collected in this work, and the experimental data on micropowder (*d_w_* = 250 μm, *δ_w_* = 900 μm and *ε_w_* = 0.64) are taken from Weibel *et al*. [24]. Model prediction based on Sudhakar *et al*. [27] for our sample s2 measurement is also presented. A simplified model, RM1 (with *h_e_* = 0 and $\eta {{q^{\prime\prime}\!\!}_t}$ = 0.9), is developed based on our model by setting consistent conditions with the assumptions of Sudhakar *et al*. [27]. Another simplified model, RM2, is developed based on our model with the assumption of *h_e_* = 0. The heat flux fraction ${{q^{\prime\prime}\!\!}_t}/{{q^{\prime\prime}\!\!}_{CHF}}$ is adopted since the CHF is varied for different microstructures in the literature. (B) The fraction of the evaporation heat flux atop the wick to the total heat flux ${{q^{\prime\prime}\!\!}_e}/{{q^{\prime\prime}\!\!}_t}$ as a function of heat flux fraction ${{q^{\prime\prime}\!\!}_t}/{{q^{\prime\prime}\!\!}_{CHF}}$. (C) Comparison of the model-predicted CHF with the experimental data and predictions from other models. The red solid line, purple triangle, and blue diamond represent our experimental result of sample s2, model prediction from Sudhakar *et al*. [27], and model prediction from Zhang *et al*. [26], respectively. (D) Comparison of measured HTC on micro-pillared surface with the predictions of this work and from pool boiling models [32,45]. (E) Comparison of measured CHF on micro-pillared surface with the predictions from this work and pool boiling models [10,46,47]. The red circles in (D) and the red solid line in (E) are the experimental data on micropillar (*d_w_* = 60 μm, *δ_w_* = 320 μm and *ε_w_* = 0.6) from Cai and Bhunia [25].

Figure 4A compares the experimental data on micropowder surface (*d_w_* = 250 μm, *δ_w_* = 900 μm and *ε_w_* = 0.64) from Weibel *et al*. [24], our experimental data on micromesh of sample s2 (*d_w_* = 50 μm, *s_w_* = 160 μm, *δ_w_* = 264 μm and *ε_w_* = 0.71), and our model predictions for HTC using Eq. (24) on the same sample. The model-predicted HTC from the Sudhakar *et al*. model [27] for our micromesh sample s2 is also presented in Fig. 4A. It is shown that the model by Sudhakar *et al*. [27] predicts a constant HTC, due to its underlying assumption of a constant evaporative area inside the micropore for nucleate boiling and adiabatic condition atop the wick (i.e. *h_e_* = 0 due to the neglect of evaporation atop the wick). In contrast, our model can predict the increasing trend of HTC with increasing heat flux, which agrees well with results from previous liquid film boiling experiments [16,18,20–24]. This is because our model considers both the evaporation atop the wick and the variation in the nucleate boiling inside the wick under varying heat flux.

To validate our model and understand the coupling roles between evaporation atop the wick and nucleate boiling inside, we develop two simplified models (RM) based Eq. (24). RM1 is a simplification with the approximations of Sudhakar *et al*. [27] with *h_e_* = 0, *δ_l_* = *δ_w_* and $\eta {{q^{\prime\prime}\!\!}_t}$= 0.9, and RM2 is a reduced model by setting *h_e_* = 0 (neglecting evaporation atop the wick). The value $\eta {{q^{\prime\prime}\!\!}_t}$= 0.9 in RM1 is derived from the assumption according to Ref. [27], where evaporation occurs uniformly at the entire liquid-vapor interface (i.e. *ϕ_ml_* = 2*π*), and the microlayer thickness is assumed as 0.1 times the effective pore radius (i.e. *δ_ml_* = 0.1*r_eff_*), giving $\eta {{q^{\prime\prime}\!\!}_t}$= *A_ml_$/$A_total_* = [(*r_eff_* − *δ_ml_*)*ϕ_ml_*]/(2*πr_eff_*)  = 0.9. As shown in Fig. 4A, RM1 can predict nearly identical results with Ref. [27], and a constant HTC is obtained. At low heat flux, the predictions of HTC from RM2 deviate from our original model. This is because at low heat flux, the heat transfer fraction of evaporation atop the wick is relatively high and RM2 neglects the contribution of the evaporation atop the wick. In our model, the relationship between evaporation atop the wick and nucleate boiling inside is self-consistent. The heat dissipation fraction of evaporation atop (${{q^{\prime\prime}\!\!}_e}/{{q^{\prime\prime}\!\!}_t}$) from our model decreases as the heat flux fraction (${{q^{\prime\prime}\!\!}_t}/{{q^{\prime\prime}\!\!}_{CHF}}$) increases (Fig. 4B). The evaporation heat dissipation fraction exceeds 20% when heat flux fraction (${{q^{\prime\prime}\!\!}_t}/{{q^{\prime\prime}\!\!}_{CHF}}$) is below 30%, which indicates that evaporation atop the wick should be considered in liquid film boiling heat transfer modeling, and its neglection may cause a large deviation, especially for low heat fluxes.

As shown in Fig. 4C, our model also exhibits good agreement with experimental data of sample s2 when predicting CHF. To further validate our model prediction, we develop RM3 based on our CHF relation of Eq. (25) by neglecting the friction of substrate (i.e. liquid wicking velocity is uniform along the *z*-direction at each *x* with $\partial {{u}_l}/\partial z = 0$) and neglecting the gravitational effect (i.e. *g* = 0), to be consistent with the case in Ref. [27]. Notably, the CHF prediction from RM3 is very close to the model by Sudhakar *et al.* [27]. Besides, a simplified model (RM4) is developed with $\partial {{u}_l}/\partial z = 0$, *g* = 0, and *K_rl_* = 0.15, according to Zhang *et al*. [26] and, as expected, the prediction is consistent.

Here, we also compare our model on liquid film boiling with the models developed for pool boiling. Since most pool boiling models are developed based on the micropillar, we select micropillars as the micro-structured surface to compare the predictions. The micropillar used in the modeling has the following geometric parameters: a pillar diameter of 60 μm, a porosity of 0.6, a thickness of 320 μm, and a wicking length of 2 mm, which are same as those used in the experiment conducted by Cai and Bhunia [25]. As shown in Fig. 4D, our model prediction for HTC shows better agreement with the experiment compared to the HTC models developed for pool boiling in literature [32,45]. This is because natural convection contributes significantly to pool boiling heat transfer, but is not as important in liquid film boiling due to the thinness of the capillary film. Although there are many CHF models for pool boiling that consider surface properties and liquid wicking [10,46,47], they are not suitable for predicting CHF in liquid film boiling (Fig. 4E), due to the different mechanisms aforementioned. For pool boiling, the relatively large bubbles easily coalesce to form a vapor blanket, leading to the thermal-hydraulic CHF. However, in liquid film boiling CHF occurs because of surface dry-out, which is caused by capillary wicking failure.

### Model prediction

In this section, we use our model to predict both HTC and CHF with unified microlayer area factor *η* = 2.15 × 10^−3^ cm^2^ W^−1^ for liquid film boiling on various types of uniform micro-structured surfaces. The wicking structures include silicon micropillar arrays [25,48], packed copper micropowders [21,24], and sintered copper micromeshes [16,23] (Table 1). The liquid supply methods for these experiments include one-side, two-side, and all-around, with a heated area ranging from 4 to 100 mm^2^, a wick thickness ranging from 200 to 1200 μm, and a wick porosity ranging from 0.51 to 0.76.

**Table 1.** Summary of the experimental conditions and sample characteristics in the literature.

Structure type	Liquid supply method	Heater area (mm^2^)	Thickness (μm)	Porosity	References
Si micropillar	One-side	4 & 16	220–320	0.60	Cai and Bhunia [25]
Si micropillar	One-side	4 & 16	320	0.60	Cai and Bhunia [48]
Cu micropowder	One-side	25	600–1200	0.63–0.65	Weibel *et al*. [24]
Cu micropowder	All-around	100	200–400	0.61–0.63	Sudhakar *et al*. [21]
Cu micromesh	One-side	100	235–370	0.51–0.54	Wen *et al*. [16]
Cu micromesh	All-around	64	370–740	0.69–0.70	Li *et al*. [23]
Cu micromesh	One-side	100	261–377	0.57–0.76	This work

**Figure 5.**
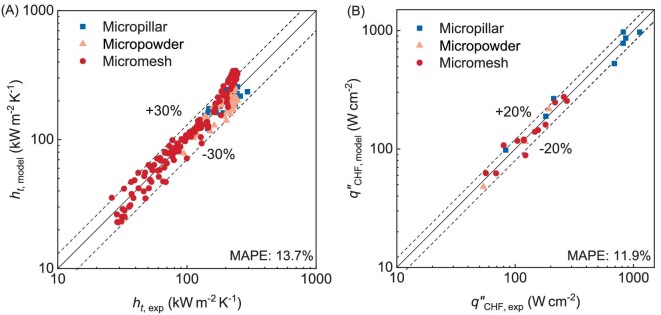
Comparison between model predictions and experimental data on various types of wicking structures: micropillar [25,48], micropowder [21,24], and micromesh [16,23]. (A) *h_t_*,_model_  *vs. h_t_*,_exp_. (B) ${{q^{\prime\prime}\!\!}_{CHF,{\mathrm{ model}}}}$  *vs.*  ${{q^{\prime\prime}\!\!}_{CHF, \exp }}$.

Figure 5A shows that the HTC predictions from our model are in good agreement with experimental results for various uniform wicking structures, achieving a MAPE of 13.7%. For the HTC ranging from 30–300 kW m^−2^ K^−1^, the model predicts the experimental data well, with an accuracy of ±30% due to its ability to calculate the heat dissipated by both nucleate boiling inside the wick and evaporation atop the wick. The experimental CHF with a range of 40–1200 W cm^−2^ could also be well predicted by our model with an error of 20% (Fig. 5B). The MAPE for CHFs of all samples is calculated to be 11.9%. We note that HTC and CHF are affected by many aspects, including surface geometries, thermal conductivity, liquid supply methods, surface wettability, and total heat flux input. Our model has taken these aspects into account and experimentally determines a factor *η* to model the variation in microlayer area fraction during liquid film boiling. Therefore, our model can predict both the HTC and CHF for various wicking structures within a spread of ±30%.

## CONCLUSION

In this work, a heat transfer model for predicting both the CHF and HTC is developed for liquid film boiling on various micro-structured surfaces. The model accounts for both evaporation atop the wick and nucleate boiling inside the wick. Evaporation atop the wick is calculated by analyzing the thermal resistance network at the thin-film region. Nucleate boiling is modeled using microlayer evaporation theory with an empirical factor *η* to describe the relationship between microlayer area fraction and heat flux as *A_ml_/A_total_* = *η*${{q^{\prime\prime}\!\!}_t}$. The scaling factor *η* is found to be independent of structural parameters and the value is determined to be *η* = 2.15 × 10^−3^ cm^2^ W^−1^ by our liquid film boiling experiments. Our model can be reduced to previous models with simplifications. With the same value of *η*, the model predictions of both HTC and CHF are in good agreement with the reported experimental data for various uniform micro-structured surfaces, including micropillar, micropowder, and micromesh, with a spread of ±30%. This work provides a tool for designing micro-structured surfaces for advancing thermal management applications.

## Supplementary Material

nwae090_Supplemental_File
