# Supplementary material for: A heat transfer model for liquid film boiling on micro-structured surfaces
Source: Natl Sci Rev. 2024 Mar 8;11(5):nwae090. doi: 10.1093/nsr/nwae090 (PMC11020273; doi:10.1093/nsr/nwae090)
Supplement: nwae090_Supplemental_File [file nwae090_supplemental_file.pdf]

Supplementary Data for

A heat transfer model for liquid film boiling on micro-structured surfaces

Pengkun Li[1], Qifan Zou[1], Xiuliang Liu[1,*] and Ronggui Yang[1,2,*]

[1]School of Energy and Power Engineering, Huazhong University of Science and Technology, Wuhan 430074, China

[2]State Key Laboratory of Coal Combustion, Huazhong University of Science and Technology, Wuhan, 430074, China

* Correspondence author: liuxiuliang@hust.edu.cn (X. Liu); ronggui@hust.edu.cn (R.G. Yang)

Table of contents

Note S1. Physical properties of wicking structures 3

Note S2. Effects of liquid supply methods on liquid wicking performance 4

Note S3. Liquid saturation s 7

Note S4. Liquid film thickness δ_l and liquid meniscus curvature H 9

Note S5. Evaporation atop the wick 12

Note S6. Heat transfer coefficient of nucleate boiling inside the micro-pillared wick 14

Note S7. Comparison of capillary evaporation and liquid film boiling on wicking structures 16

Note S8. Theoretical analysis of the microlayer area factor η 17

Note S9. Sample fabrication, wetting characterization and liquid wicking experiment 19

Note S10. Experimental setup, uncertainty analysis and repeatability for liquid film boiling 21

Nomenclature

A	area (m^2)	*Greek symbol*	
A_{xy}	projected area in the *x*-*y* plane	δ	thickness (m)
c_f	compression factor	Δ	difference
C_E	Ergun coefficient	ε	porosity
d	wire/particle/pillar diameter (m)	μ	dynamic viscosity (kg m^{-1} s^{-1})
D_w	equivalent diameter of wick (m)	ρ	density (kg m^{-3})
F	Force (N)	σ	surface tension (N m^{-1})
h	heat transfer coefficient (W $m^{-2}K^{-1}$)	σ_a	accommodation coefficient
h_w	wicking height (m)	θ	contact angle (°)
H	liquid meniscus curvature (m^{-1})	θ_R	receding contact angle (°)
h_{fg}	specific latent heat (kJ kg^{-1})		
k	thermal conductivity (W $m^{-1}K^{-1}$)	*Subscripts*	
K	permeability (m^2)	*ad*	adsorbed liquid film
l	length	*avg*	averaged
L	wicking length (m)	*b*	nucleate boing inside the wick
M	mesh number	*c*	capillary
$\bar{M}$	molar mass (kg mol^{-1})	*CHF*	critical heat flux
$\dot{m}$	mass flow rate (kg s^{-1})	*e*	evaporation atop the wick
p	central distance of wires/particles/pillars (m)	*eff*	effective
P	pressure (Pa)	*i*	liquid-vapor interface
p_l	distance between tri-phase lines (m)	*l*	liquid
q''	heat flux (W m^{-2})	*max*	maximum
r	radius (m)	*ml*	microlayer
r_c	characteristic radius of wick solid skeleton (m)	*rl*	relative liquid
R	thermal resistance (K W^{-1})	*rv*	relative vapor
R_u	universal gas constant (J $mol^{-1}K^{-1}$)	*s*	solid
s	liquid saturation	*sat*	saturated
s_w	spacing width of mesh	*t*	total
S_v	surface area per unit volume of solid (m^{-1})	*tf*	thin-film
t	time (s)	*tp*	tri-phase line
T	temperature (°C)	*unit*	unit cell of wicking structure
u	velocity (m s^{-1})	*v*	vapor
x	wicking distance (m)	*w*	wicking structure
z	distance from the heated surface (m)	*z*	component in the *z*-direction

Note S1. Physical properties of wicking structures

To compare model predictions with literature data, we study three typical wicking structures in the literature, including micromesh [1], micropowder [2], and hexagonal micropillar array [3]. The effective thermal conductivity and effective pore radius for different wicking structures are estimated using the correlations reported in the literature, which are summarized in Table S1. To consider the curved liquid meniscus on the wick permeability, the absolute permeability K_w of the wick is estimated [4] as

$$K_w = K_{2D}\left[1-\frac{\tanh\left(\sqrt{\frac{\varepsilon_w}{K_{2D}}}\delta_l\right)}{\sqrt{\frac{\varepsilon_w}{K_{2D}}}\delta_l}\right], \tag{S1}$$

where K_{2D} is the uniform permeability for wicking structures without substrate friction. The correlations for calculating K_{2D} are also summarized in Table S1.

Table S1. Calculation of wick porosity ε_w [5, 6], uniform permeability K_{2D} [7-9], effective pore radius r_{eff} [2, 10, 11], effective thermal conductivity k_{eff} [3, 5, 12], and characteristic radius of wick solid skeleton r_c [13].

Wick type	Micromesh	Micropowder	Hexagonal micropillar array
Porosity ε_w	$1-\frac{\pi M d_w\sqrt{1+(Md_w)^2}}{4c_f}$	$1-\frac{\pi\left(\frac{d_w}{2}\right)^2}{p_w^2}$	$1-\frac{2\sqrt{3}\pi}{3}\frac{\left(\frac{d_w}{2}\right)^2}{p_w^2}$
Uniform permeability K_{2D}	$\frac{{d_w}^2\varepsilon_w^3}{122(1-\varepsilon_w)^2}$	$\frac{{d_w}^2\varepsilon_w^3}{240(1-\varepsilon_w)^2}$	$\frac{p_w^2\ln\left[\sqrt{\frac{2\sqrt{3}}{\pi}\frac{p_w}{d_w}}-0.745+\frac{\pi}{2\sqrt{3}}\left(\frac{d_w}{p_w}\right)^2-\frac{\pi^2}{48}\left(\frac{d_w}{p_w}\right)^4\right]}{4\pi}$
Effective pore radius r_{eff}	$\frac{\sqrt{{p_w}^2+(d_wc_f)^2}-d_wc_f}{2}$	$0.21d_w$	$\frac{\varepsilon_w d_w}{2(1-\varepsilon_w)}$
Effective thermal conductivity k_{eff}	$\frac{1.42(Md_w)^2}{c_f}k_s$	$\frac{2+k_l/k_s-2\varepsilon_w(1-k_l/k_s)}{2+k_l/k_s+\varepsilon_w(1-k_l/k_s)}k$	$(1-\varepsilon_w)k_s$
Characteristic radius of solid skeleton r_c	$\frac{d_w}{2}$	$\frac{d_w}{2}$	$\frac{d_w}{2}$

Note S2. Effects of liquid supply methods on liquid wicking performance

Two-side liquid supply enhances the liquid rewetting capability of heated surface compared to one-side liquid supply. Assuming that liquid supply in the two-side liquid supply is symmetrical (Fig. S1A), the boundary condition for the mass flow rate of liquid can be expressed as:

$$\dot{m}_l(x)\Big|_{x=0} = \rho_l \bar{u}_l(x)\delta_l(x)\Big|_{x=0} = \frac{q_t''}{\rho_l h_{fg}}\frac{L}{2}. \tag{S2}$$

Combining this equation with Eq. (11) in the main text, $\bar{u}_l(x)$ can be obtained as:

$$\bar{u}_l(x) = \frac{q_t''}{2\rho_l h_{fg}}\frac{(L-2x)}{\delta_l(x)}. \tag{S3}$$

Combing Eq. (S3) and Eq. (10) in the main text, the pressure gradient $\mathrm{d}P_l/\mathrm{d}x$ can be obtained as

$$\frac{\mathrm{d}P_l}{\mathrm{d}x} = -\frac{\mu_l q_t''}{2\rho_l h_{fg}K_w}\frac{(L-2x)}{K_{rl}\delta_l\left[1-\frac{\tanh(\lambda\delta_l)}{\lambda\delta_l}\right]}. \tag{S4}$$

The CHF of liquid film boiling occurs when liquid pressure drop is equal to the maximum capillary pressure $P_{c,\max}$ in the wick [11], i.e., $-\int_0^{L/2}(\mathrm{d}P_l/\mathrm{d}x)\,\mathrm{d}x = P_{c,\max}$. Integrating Eq. (S4) from $x=0$ to $x=L/2$, CHF can then be obtained as:

$$q_{CHF}'' = \frac{1}{\mu_l}\frac{2\rho_l h_{fg}K_w P_{c,\max}}{\int_0^{\frac{L}{2}}\frac{(L-2x)}{K_{rl}\delta_l\left[1-\tanh(\lambda\delta_l)/(\lambda\delta_l)\right]}\mathrm{d}x}. \tag{S5}$$

When neglecting substrate friction as $\partial u_l/\partial z = 0$, assuming uniform vapor distribution as K_{rl} = const and assuming liquid film thickness to be the wick thickness as $\delta_l \approx \delta_w$, Eq. (S5) reduces to $q_{CHF}'' = \frac{8P_{c,\max}\rho_l h_{fg}\delta_w K_{rl}K_w}{\mu_l L^2}$, which is same as the correlation in ref. [14].

As shown in Fig. S1B, for the liquid wicking inside a wicking structure with the all-around liquid supply, when gravity is neglected, the momentum equation governing the liquid flow inside the wicking structures can be expressed by the Brinkman equation [15], as

$$\frac{\partial^2 u_l(x,z)}{\partial z^2} + \frac{1}{x}\frac{\partial}{\partial x}\left(x\frac{\partial u_l(x,z)}{\partial x}\right) - \frac{\varepsilon_l u_l(x,z)}{K_{rl}K_w} - \frac{\varepsilon_l}{\mu_l}\frac{\mathrm{d}P_l}{\mathrm{d}x} = 0, \tag{S6}$$

with the boundary conditions:

$$\left. u_l(x,z) \right|_{z=0} = 0, \quad \left. \frac{\partial u_l(x,z)}{\partial z} \right|_{z=\delta_l} = 0. \tag{S7}$$

For the thin liquid film inside the wick, it is reasonable to assume that $\frac{\partial^2 u_l(x,z)}{\partial z^2} \gg \frac{1}{x}\frac{\partial}{\partial x}\left(x\frac{\partial u_l(x,z)}{\partial x} \right)$ due to a much larger heating area than wicked liquid film thickness as $L \gg \delta_w$. Eq. (S6) can then be simplified as:

$$\frac{\partial^2 u_l(x,z)}{\partial z^2} = \frac{\varepsilon_l u_l(x,z)}{K_{rl}K_w} + \frac{\varepsilon_l}{\mu_l}\frac{\mathrm{d}P_l}{\mathrm{d}x}, \tag{S8}$$

and pressure gradient $\mathrm{d}P_l/\mathrm{d}x$ can then be obtained as

$$\frac{\mathrm{d}P_l}{\mathrm{d}x} = -\frac{\mu_l \bar{u}_l(x)}{K_{rl}K_w\left(1 - \frac{\tanh(\lambda\delta_l)}{\lambda\delta_l}\right)}. \tag{S9}$$

Based on the energy balance, the average liquid wicking velocity can also be written as

$$\bar{u}_l(x) = \frac{q_t'' \pi x^2}{\rho_l h_{fg} \delta_l (2\pi x)}. \tag{S10}$$

Combing Eqs. (S9) and (S10), the pressure gradient $\mathrm{d}P_l/\mathrm{d}x$ can be reformulated as:

$$\frac{\mathrm{d}P_l}{\mathrm{d}x} = -\frac{\mu_l q_t'' x}{2\rho_l h_{fg} K_w} \frac{1}{K_{rl}\delta_l\left[1 - \frac{\tanh(\lambda\delta_l)}{\lambda\delta_l}\right]}. \tag{S11}$$

The CHF of liquid film boiling occurs when liquid pressure drop is equal to the maximum capillary pressure $P_{c,\max}$ in the wick [11], i.e., $-\int_{L/2}^{0} (\mathrm{d}P_l/\mathrm{d}x)\,\mathrm{d}x = P_{c,\max}$. Integrating Eq. (S11) from $x = L/2$ to $x = 0$, CHF can then be obtained as:

$$q''_{CHF} = \frac{1}{\mu_l} \frac{2\rho_l h_{fg} K_w P_{c,\max}}{\int_{L/2}^{0} \frac{x}{K_{rl}\delta_l\left[1 - \tanh(\lambda\delta_l)/(\lambda\delta_l)\right]}\mathrm{d}x}. \tag{S12}$$

When neglecting substrate shear as $\partial u_l/\partial z = 0$, assuming uniform vapor distribution as K_{rl}

= const and simplifying liquid film thickness to be wick thickness as $\delta_l \approx \delta_w$, Eq. (25) in the main text for one-side liquid supply reduces to $q''_{CHF} = \frac{2(P_{c,\max} - \rho_l g L)\rho_l h_{fg} \delta_w K_{rl} K_w}{\mu_l L^2}$, Eq. (S5) for two-side liquid supply reduces to $q''_{CHF} = \frac{8P_{c,\max}\rho_l h_{fg} \delta_w K_{rl} K_w}{\mu_l L^2}$, and Eq. (S12) for all-around liquid supply reduces to $q''_{CHF} = \frac{16P_{c,\max}\rho_l h_{fg} \delta_w K_{rl} K_w}{\mu_l L^2}$. From these equations, we can clearly see that the all-around liquid supply can significantly increase the CHF compared to other two liquid supply methods.

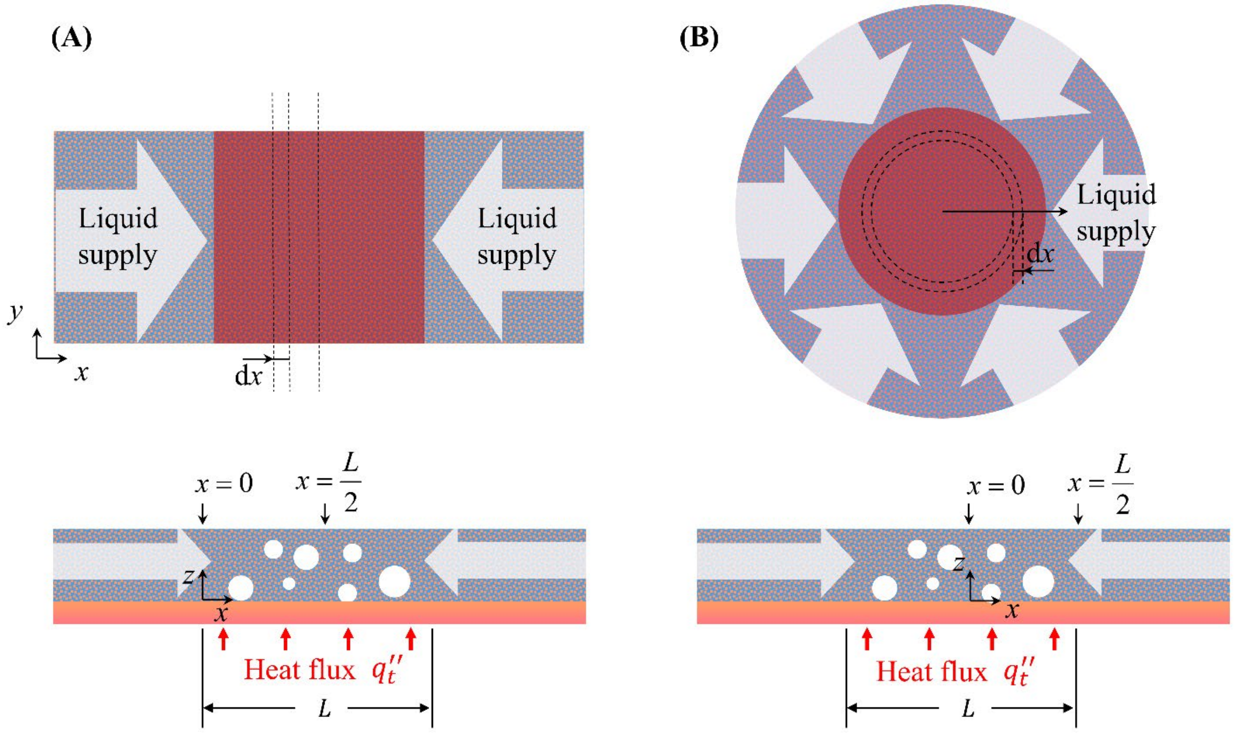

Figure S1. Schematic of capillary-driven liquid film boiling with (A) two-side and (B) all-around liquid supply.

Note S3. Liquid saturation *s*

The effective liquid permeability K_{rl} is a complex function of many parameters related to two-phase fluids and wicking structure, such as phase saturation and contact angle [16]. Previous studies have shown that K_{rl} for boiling inside the wick has low sensitivity to other parameters, except liquid saturation s [3, 14, 16, 17]. Therefore, the relative permeability is usually written as a power function of the liquid saturation s as [16]

$$\begin{aligned} K_{rl} &= s^3, \\ K_{rv} &= (1-s)^3, \end{aligned} \tag{S13}$$

where K_{rv} is effective vapor permeability, which can be determined by analyzing the vapor flow. The vapor flow in the z-direction is driven by the decreased vapor pressure along the z-direction, which can be expressed by the modified Ergun equation [18]

$$\frac{\mathrm{d}P_v}{\mathrm{d}z} = \frac{\mu_v}{K_{rv}K_w}u_v + \frac{C_E(1-\varepsilon_v)\rho_v}{\varepsilon_v^3 D_w}u_v^2, \tag{S14}$$

where P_v is the vapor pressure, μ_v and ρ_v are dynamic viscosity and density of vapor, respectively. Here, D_w is the equivalent diameter of wick estimated by $D_w = 6/S_v$ with S_v being the surface area per unit volume of solid phase [18], $\varepsilon_v = (1 - s)\varepsilon_w$ is the effective porosity for vapor, u_v is the vapor flow velocity calculated by energy balance, as $u_v = q_b'' / \rho_v h_{fg}$, q_b'' is the heat flux of the nucleate boiling calculated as $q_b'' = q_t'' - q_e''$. By integrating Eq. (S14) with the boundary condition $P_v|_{x=\delta_l} = P_{sat}$, the vapor pressure P_v can be derived as

$$P_v(z) = P_{sat} + \left(\frac{\mu_v}{K_{rv}K_w}\frac{q_b''}{\rho_v h_{fg}} + C_E \frac{1-(1-s)\varepsilon_w}{(1-s)^3\varepsilon_w^3}\frac{1}{D_w}\frac{q_b''^2}{\rho_v h_{fg}^2} \right)(z-\delta_l). \tag{S15}$$

The average vapor pressure can be obtained as

$$P_{v,avg} = \frac{1}{\delta_l}\int_0^{\delta_l} P_v(z)\mathrm{d}z = P_{sat} + \frac{C}{(1-s)^2} + \frac{D}{(1-s)^3}, \tag{S16}$$

where $C = -\frac{\delta_l C_E}{2\varepsilon_w^2 D_w}\frac{q_b''^2}{\rho_v h_{fg}^2}$ and $D = \frac{\delta_l}{2}\left(\frac{\mu_v}{K_w}\frac{q_b''}{\rho_v h_{fg}} + \frac{C_E}{\varepsilon_w^3 D_w}\frac{q_b''^2}{\rho_v h_{fg}^2} \right)$.

In the bubble boundary, force balance dictates that vapor pressure $P_{v,avg}$ equals the sum of the liquid pressure P_l and the Laplace pressure $P_{c,b}$ at the liquid-vapor interface between the bubble

and the liquid, namely, $P_{v,avg} = P_l + P_{c,b}$. In liquid film boiling, $P_{c,b}$ can be expressed as a function of liquid saturation s as [19]: $P_{c,b} = P_{c,\max}(1\text{-}s)$. By substituting this equation into Eq. (S16), the equation to calculate liquid saturation s is obtained as

$$P_{c,\max}\left(1-s\right)^4-\left(P_l-P_{sat}\right)\left(1-s\right)^3-C(1-s)-D=0. \tag{S17}$$

From Eq. (S17), we can see that the liquid saturation is a function of the liquid pressure. The relative liquid permeability K_{rl} as a function of liquid pressure P_l can then be obtained by combining Eqs. (S13) and (S17). When solving Eq. (14) in the main text, we substitute Eqs. (13) and (S17) into Eq. (14), transforming Eq. (14) into an ODE with the sole variable P_l.

Note S4. Liquid film thickness δ_l and the curvature of liquid meniscus H

We assume that the meniscus shape atop the wick is spherical. For micropillar, the varying contact angles ranging from receding contact angle θ_R to $\pi/2$ and const liquid film thickness are usually used due to the contact line pinning effect [20]. For micromesh and micropowder, constant receding contact angle θ_R and varying liquid film thickness δ_l are usually used. To calculate the liquid film thickness, the liquid meniscus curvature H should be determined, which is related to the capillary pressure by the Young-Laplace equation as:

$$H = \frac{P_v - P_l}{2\sigma} = \frac{P_c}{2\sigma}, \tag{S18}$$

As shown in Fig. S2, the capillary force is determined by applying force balance on the curved liquid meniscus atop the wick [21], as:

$$P_c = \Delta P = \frac{F_{cz}}{A_{xy}} = \frac{\sigma l_{tp} \sin\omega}{A_{xy}}, \tag{S19}$$

where P_c is the capillary force, F_{cz} is the component of surface tension in the z-direction, A_{xy} is the projected area of the liquid-vapor interface in the x-y plane, l_{tp} is the length of the tri-phase line of one unit cell, and ω is the angle between the surface tension direction and the horizontal direction. Combing Eqs. (S18) and (S19), the liquid meniscus curvature H is obtained as:

$$H = \frac{1}{2}\frac{l_{tp}}{A_{xy}}\sin\omega, \tag{S20}$$

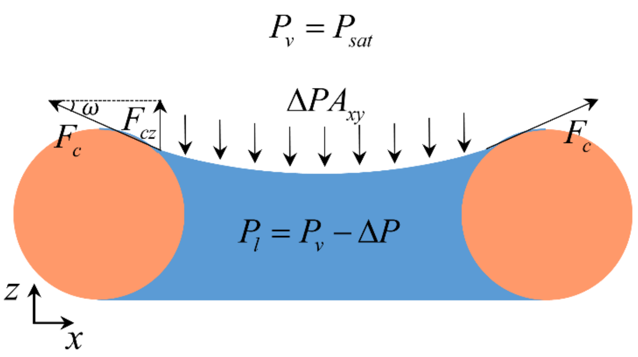

Figure S2. Schematic of force acting on the top surface of wick.

As shown in Fig. S3, different wicking structures are analyzed in this work. We note that l_{tp} represents the length of the red solid line in Fig. S3, while A_{xy} corresponds to the area of the red polygon. l_{tp} and A_{xy} as the function of ω for different structures are summarized in Table S2.

The relationship between liquid pressure P_l and liquid film thickness δ_l is used for calculating Eq. (14) in the main text. For a given liquid pressure P_l, the curvature of liquid meniscus H is obtained with Eq. (S18). The angle ω can then be obtained from Eq. (S20) and the meniscus shape can then be determined. We can then obtain the maximum and minimum liquid film thickness. The averaged liquid film thickness δ_l is used in our model, with equations summarized in Table S3. Besides, the maximum capillary pressure $P_{c,\max}$ can be determined with Eq. (S19) accordingly. The equations for calculating $P_{c,\max}$ are also summarized in Table S3 and used to calculate the CHF of Eq. (24) in the main text.

Table S2. Equations of l_{tp} and A_{xy} for calculating liquid meniscus curvature H.

Wick type	Micromesh	Micropowder	Hexagonal micropillar array
l_{tp}	$2\left(p_w - p_l\right)\left(1+\frac{p_w}{d_w}\right)\sin\omega$	$\pi\left(p_w - p_l\right)\sin\omega$	$\pi\frac{d_w}{2}$
A_{xy}	$p_w^2 - \left(p_w - p_l\right)^2 \frac{p_w}{d_w}$	$p_w^2 - \pi\left(\frac{p_w - p_l}{2}\right)^2$	$\frac{\sqrt{3}}{4}p_w^2 - \frac{\pi}{2}\left(\frac{d_w}{2}\right)^2$
p_l	$p_w - d_w\sin\left(\omega+\theta\right)$	$p_w - d_w\sin\left(\omega+\theta\right)$	

Table S3. Equations for liquid film thickness δ_l and the maximum capillary pressure $P_{c,\max}$.

Wick type	Micromesh	Micropowder	Hexagonal micropillar array
$\delta_{l,\max}$	$\delta_w - \frac{d_w}{2}\left[1-\cos\left(\theta+\omega\right)\right]$	$\delta_w - \frac{d_w}{2}\left[1-\cos\left(\theta+\omega\right)\right]$	δ_w
$\delta_{l,\min}$	$\delta_w - \frac{d_w}{2}\left[1-\cos\left(\theta+\omega\right)\right] - r_{tf}\left(1-\cos\omega\right)$	$\delta_w - \frac{d_w}{2}\left[1-\cos\left(\theta+\omega\right)\right] - r_{tf}\left(1-\cos\omega\right)$	$\delta_w - r_{tf}\left(1-\cos\omega\right)$
δ_l	$\delta_w - \frac{d_w}{2}\left[1-\cos\left(\theta+\omega\right)\right] - \frac{r_{tf}}{2}\left(1-\cos\omega\right)$	$\delta_w - \frac{d_w}{2}\left[1-\cos\left(\theta+\omega\right)\right] - \frac{r_{tf}}{2}\left(1-\cos\omega\right)$	$\delta_w - \frac{r_{tf}}{2}\left(1-\cos\omega\right)$
r_{tf}	$\frac{p_l}{2\sin\omega}$	$\frac{p_l}{2\sin\omega}$	$\frac{p_w - d_w}{2\sin\omega}$
$P_{c,\max}$	$\frac{2\sigma\left(p_w + d_w\right)}{p_w\left(p_w - d_w\right)}\cos\theta_R$	$\frac{\pi\sigma d_w}{p_w^2 - \pi\left(\frac{d_w}{2}\right)^2}\cos\theta_R$	$\frac{2\pi\sigma d_w}{\sqrt{3}p_w^2 - 2\pi\left(\frac{d_w}{2}\right)^2}\cos\theta_R$

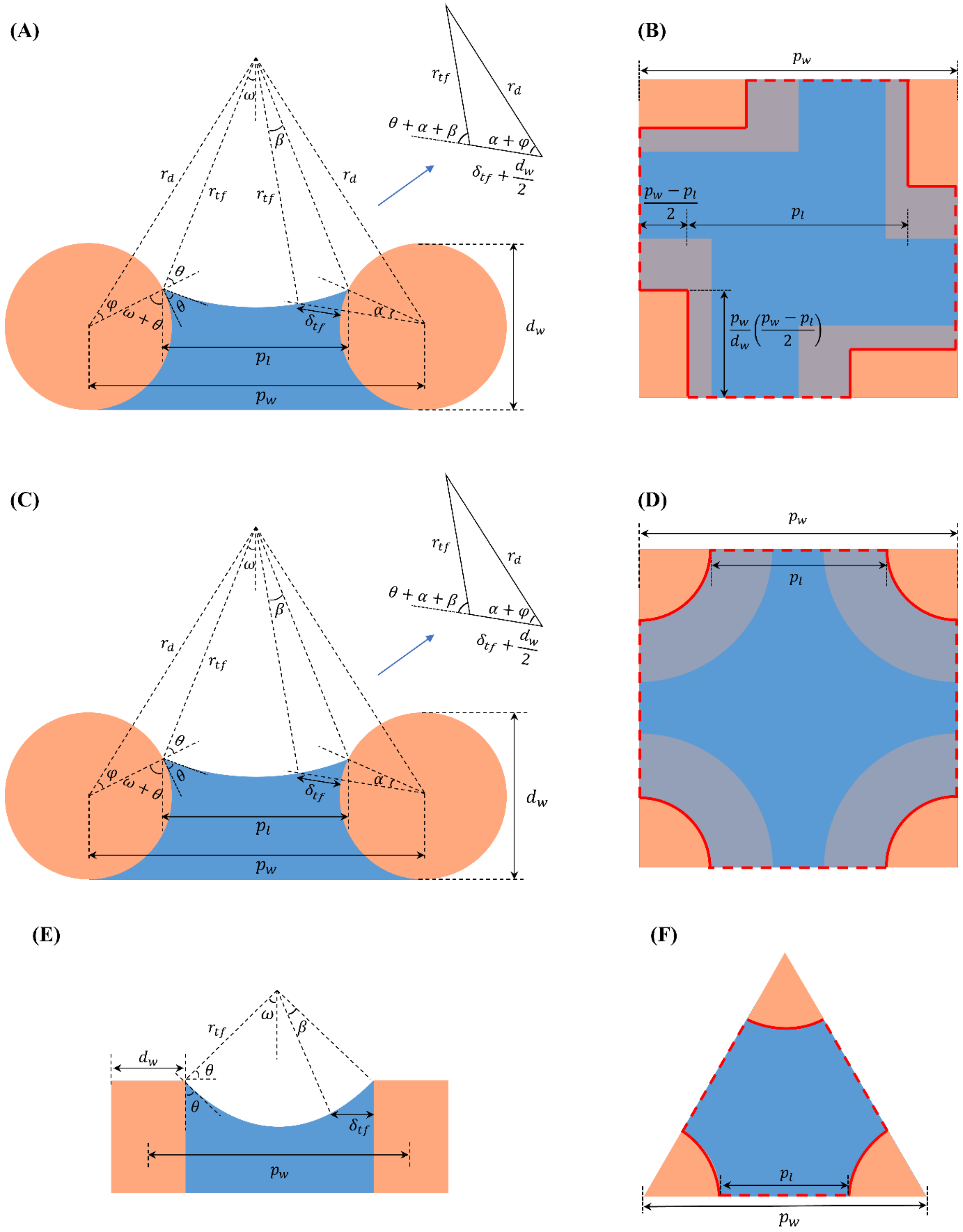

Figure S3. Schematics of the unit cell of the wicking structures. (A) Side view and (B) top view of the liquid film meniscus on micromesh surface at a specific liquid film thickness. (C) Side view and (D) top view of the liquid film meniscus on micropowder surface at a specific liquid film thickness. (E) Side view and (F) top view of the liquid film meniscus on micropillar surface at a specific liquid film thickness.

Note S5. Evaporation atop the wick

A unit cell of wick is used to analyze the heat transfer coefficient of evaporation on the top of the wicking structure. As shown in Fig. S4, the heat at the top surface is mainly dissipated by evaporation through the thin-film region [13], which is defined as the liquid film with a thickness between the adsorbed film thickness (δ_{ad}) to the maximum thin-film thickness effective for evaporation ($\delta_{tf,\max}$). The heat is first conducted through the thin liquid film with a thermal conduction resistance R_{tf}, and then dissipated through interfacial evaporation with an interfacial resistance $R_{i,e}$. R_{tf} and $R_{i,e}$ can be determined by [22]

$$R_{tf} = \left[\int_{\delta_{ad}}^{\delta_{tf,\max}} \frac{k_l}{\delta_{tf}} \mathrm{d}A_{i,e}(\delta_{tf}) \right]^{-1}, \tag{S21}$$

$$R_{i,e} = \frac{1}{h_i A_{i,e}}, \tag{S22}$$

where $A_{i,e}$ is the interfacial area of the thin-film region and δ_{tf} ranging from δ_{ad} to $\delta_{tf,\max}$ is a thin liquid film thickness at the thin-film region. The thickness δ_{tf} and the area $A_{i,e}$ of the thin-film region can be obtained from Table S4. More detailed derivation about δ_{tf} and $A_{i,e}$ can be found in our previous work [22]. h_i is the heat transfer coefficient for the evaporation at the liquid-vapor interface, which can be estimated using the Schrage equation [23], given by

$$h_i = \frac{2\sigma_a}{2-\sigma_a} \frac{h_{fg}^2}{T_{sat} v_{fg}} \sqrt{\frac{\bar{M}}{2\pi R_u T_{sat}}} \left(1 - \frac{P_{sat} v_{fg}}{2h_{fg}}\right), \tag{S23}$$

where $\sigma_a = 0.04$ for water is the accommodation coefficient [24], $\bar{M} = 0.018$ kg mol^{-1} is the molar mass of liquid, $R_u = 8.314$ J mol^{-1}K^{-1} is the universal gas constant, and v_{fg} is the specific volume between vapor and liquid phases.

From Eqs. (6), (7), and (15) in the main text, the heat flux fraction of evaporation atop the wick can then be calculated as:

$$\frac{q_e''}{q_t''} = \frac{h_e}{\sqrt{h_{bv} k_{eff}} \sinh(m\delta_l) + h_e \cosh(m\delta_l)}, \tag{S24}$$

where $h_e = [(R_{tf} + R_{i,e}) A_{unit}]^{-1}$. From Eq. (S24), it is clear that the heat transfer fraction of evaporation atop the wick depends on the heat transfer coefficients of both evaporation atop the

wick (h_e) and nucleate boiling inside (h_{bv}). By obtaining the values of h_e and h_{bv}, the heat flux of the evaporation atop the wick and the nucleate boiling inside can be determined self-consistently.

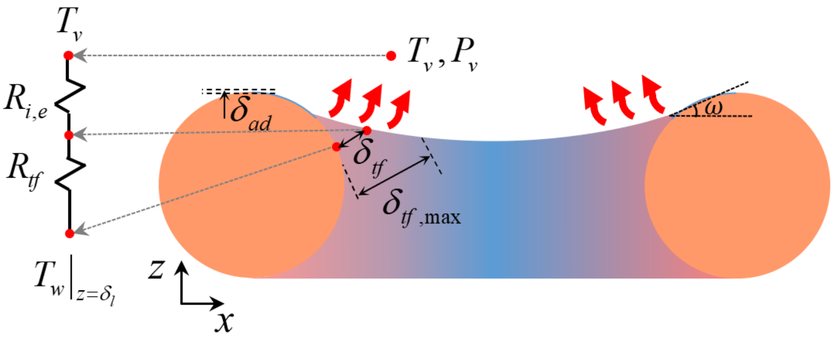

Figure S4. Schematic of evaporation at the top surface of wick.

Table S4. Geometrical parameters for liquid meniscus

Wick type	Micromesh	Micropowder*	Hexagonal micropillar array
A_{unit}	p_w^2	p_w^2	$\frac{\sqrt{3}}{4}p_w^2$
δ_{tf}	$r_d\cos(\varphi+\alpha)-\sqrt{r_{tf}^2-r_d^2\sin^2(\varphi+\alpha)}-\frac{d_w}{2}$	$r_d\cos(\varphi+\alpha)-\sqrt{r_{tf}^2-r_d^2\sin^2(\varphi+\alpha)}-\frac{d_w}{2}$	$\frac{p_w-d_w}{2}-r_{tf}\sin(\omega-\beta)$
$A_{i,e}$	$c_{wick}r_{tf}\left\{p_w(\beta_{\max}-\beta_{\min})-2r_{tf}\left[\cos(\omega-\beta_{\max})-\cos(\omega-\beta_{\min})\right]\right\}^*$		
r_d	$\sqrt{r_{tf}^2+\left(\frac{d_w}{2}\right)^2+r_{tf}d_w\cos\theta}$	$\sqrt{r_{tf}^2+\left(\frac{d_w}{2}\right)^2+r_{tf}d_w\cos\theta}$	
φ	$\arcsin\left(\frac{r_{tf}\sin\theta}{r_d}\right)$	$\arcsin\left(\frac{r_{tf}sin\theta}{r_d}\right)$	
α	$\arccos\left(\frac{(\delta_{tf}+d_w/2)^2+r_d^2-r_{tf}^2}{2(\delta_{tf}+d_w/2)r_d}\right)-\varphi$	$\arccos\left(\frac{(\delta_{tf}+d_w/2)^2+r_d^2-r_{tf}^2}{2(\delta_{tf}+d_w/2)r_d}\right)-\varphi$	
β	$\arcsin\left(\frac{r_d\sin(\varphi+\alpha)}{r_{tf}}\right)-\theta-\alpha$	$\arcsin\left(\frac{r_d\sin(\varphi+\alpha)}{r_{tf}}\right)-\theta-\alpha$	$\omega-\arcsin\left(\frac{p_w-d_w-2\delta_{tf}}{2r_{tf}}\right)$
c_{wick}	$4\left(1+\frac{p_w}{d_w}\right)$	π	$\frac{\pi}{2}$

* By substituting $\delta_{tf}=\delta_{ad}$ and $\delta_{tf}=\delta_{tf,\max}$ into the expressions of β and α, we can then obtain the expression for $\beta_{\min}$, $\alpha_{\min}$, $\beta_{\max}$, $\alpha_{\max}$, respectively.

Note S6. Heat transfer coefficient of nucleate boiling inside the micro-pillared wick

We directly analyze the micropillar unit to model the nucleate boiling heat transfer coefficient ($h_{bv,pillar}$) of the micro-pillared wicking surface. This coefficient is then compared with the h_{bv} obtained from the micro-pore unit simplification. Figure S5A illustrates a control volume with a height of Δl in the micro-pillared surface. The top view of this volume is shown in Fig. S5B. Microlayer evaporation occurs at the microlayer region with a liquid film thickness of δ_{ml}. Heat transfers through a microlayer conduction resistance R_l from the pillar wall to the liquid-vapor interface (Fig. S5C), and then evaporates to the vapor with an interfacial resistance $R_{i,b}$, given by:

$$R_l = \int_{r_p}^{r_p+\delta_{ml}} \frac{\mathrm{d}r}{k_l \phi'_{ml} r \cdot \Delta l} = \frac{1}{k_l \phi'_{ml} \Delta l} \ln\left(\frac{r_p+\delta_{ml}}{r_p}\right), \tag{S25}$$

$$R_{i,b} = \frac{1}{h_i A_{ml} \Delta l} = \frac{1}{h_i \left(r_p+\delta_{ml}\right) \phi'_{ml} \Delta l}. \tag{S26}$$

The total heat dissipated by microlayer evaporation Δq_b within the micropore can then be expressed as $\Delta q_b = (T_w - T_v)/(R_l + R_{i,b})$. Assuming the evaporation within the micropore dissipates heat with an equivalent volume-averaged heat transfer coefficient h_{bv}, Δq_b can also be expressed as $\Delta q_b = h_{bv,pillar} V_{up}(T_w - T_v)$, where $V_{up} = a \cdot b \cdot \sin\gamma \cdot \Delta l = \pi r_p^2 \Delta l / (1-\varepsilon_w)$ is the unit volume. Since $A_{total} = 2\pi\, r_p \cdot \Delta l$, V_{up} can be rewritten as $V_{up} = r_p A_{total}/2(1-\varepsilon_w)$.

By combining the two mathematical formulations of Δq_b, the volumetric heat transfer coefficient of nucleate boiling h_{bv} can then be obtained as $h_{bv,pillar} = 1/V_{up}\,(R_l + R_{i,b})$, which can be rewritten as:

$$h_{bv,\,pillar} = \frac{2\left(1-\varepsilon_w\right) k_l}{r_p\left(r_p+\delta_{ml}\right)\left(\ln\left(\frac{r_p+\delta_{ml}}{r_p}\right) + \frac{k_l}{h_i\left(r_p+\delta_{ml}\right)}\right)} \frac{A_{ml}}{A_{total}}. \tag{S27}$$

By substituting $r_{eff} = \varepsilon_w r_p/(1-\varepsilon_w) = \chi r_p$ into Eq. (S27), we then obtain:

$$h_{bv,\,pillar} = \frac{2\varepsilon_w k_l}{r_{eff}\left(r_{eff}+\chi\delta_{ml}\right)\left(\ln\left(1+\frac{\chi\delta_{ml}}{r_{eff}}\right) + \frac{k_l}{h_i\left(r_{eff}+\chi\delta_{ml}\right)}\right)} \eta q''_t. \tag{S28}$$

where $\chi = \varepsilon_w/(1-\varepsilon_w)$ for micropillar.

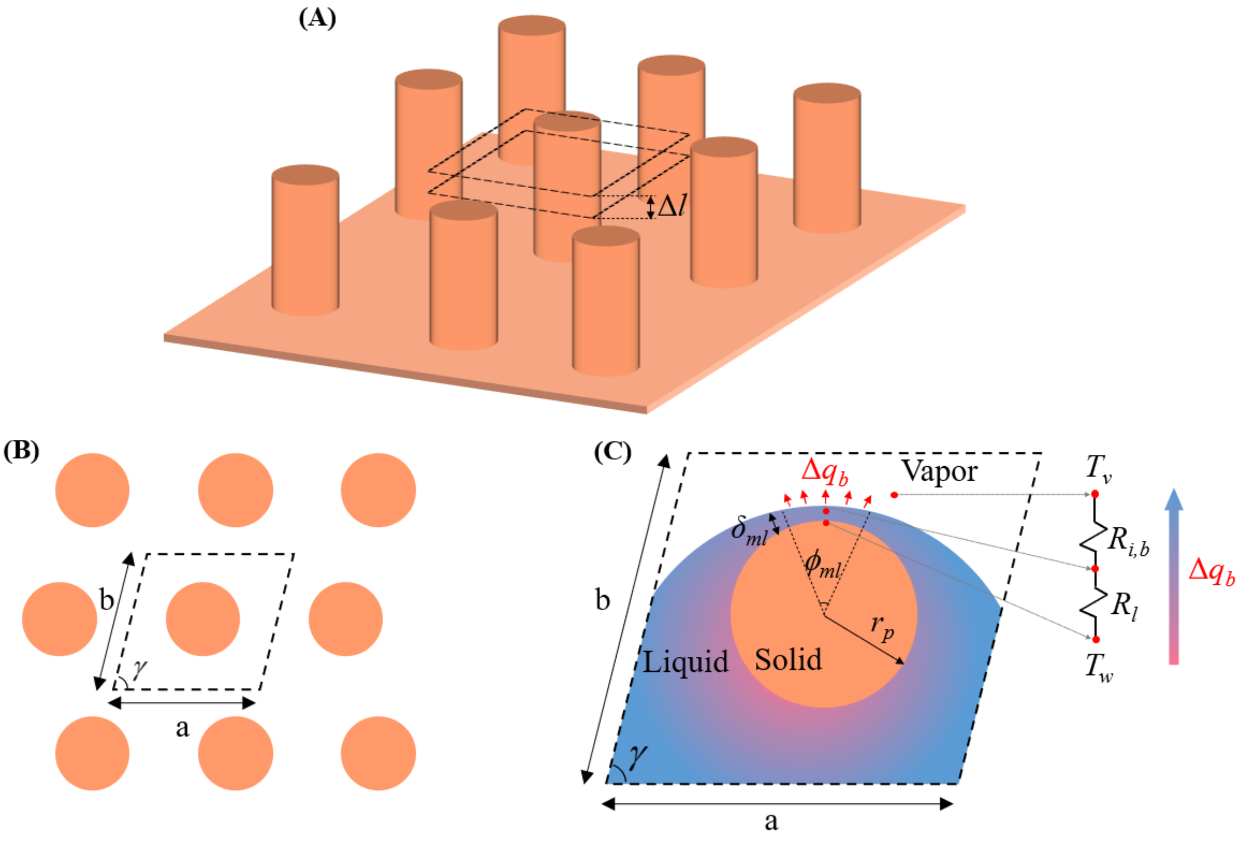

Figure S5. (A) Three-dimensional and (B) top views of the micro-pillared wicking surface, where a unit cell with a height of Δl is adopted. (C) Nucleate boiling on a unit cell is modeled as microlayer evaporation at the microlayer region with an average thickness of δ_{ml}.

Note S7. Comparison of capillary evaporation and liquid film boiling on wicking structures

To compare the heat transfer performance of capillary evaporation and liquid film boiling, we study the thickness-dependent heat transfer performance of capillary evaporation/liquid film boiling on micro-pillared surface. Here, we use micro-pillared surface as they allow for continuous variation in thickness under the constant wick porosity. Other geometry parameters for the micropillar are selected: $d_w = 60\mu m$, $\varepsilon_w = 0.8$, and $L_w = 10$ mm. For a given pillar thickness (height), we first determine if bubble nucleation can happen using our previous model [22]. If a bubble can form, we predict the heat transfer performance of liquid film boiling using the model developed in this work. If a bubble cannot nucleate, we predict the heat transfer performance of capillary evaporation using a simplified version of our model by setting $h_{bv} = 0$ and $s_v = 0$ (indicating no bubbles inside the pillar).

As shown in Fig. S6A, capillary evaporation only occurs when the wick thickness is smaller than 43.2 μm. For wick thickness ranging from 43.3 to 68.3 μm, the CHF of liquid film boiling is smaller than that of capillary evaporation on the surface with a pillar thickness of 43.2 μm. This is because the bubbles inside occupy part of the liquid transport passages and increase the liquid flow resistance. Interestingly, further increase in the pillar height results in a significant enhancement in CHF of liquid film boiling, which is much higher than that of capillary evaporation. Unlike the CHF, bubble nucleation in liquid film boiling leads to a significant improvement in HTC due to the expansion of the evaporative area (Fig. S6B).

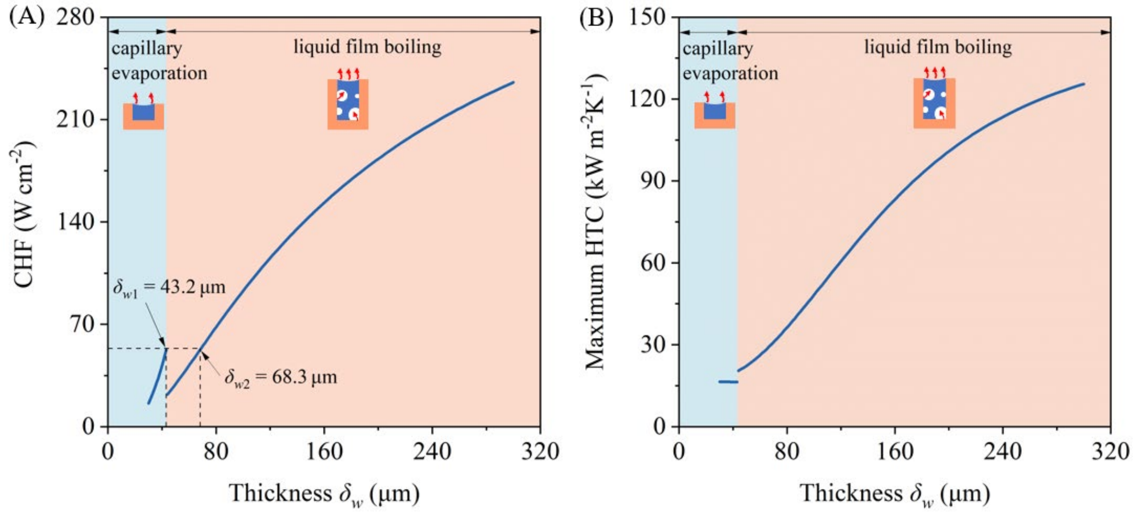

Figure S6. (A) CHF and (B) maximum HTC (HTC at CHF) as the function of wick thickness δ_w on micro-pillared surface ($d_w = 60\mu m$, $\varepsilon_w = 0.8$ and $L_w = 10$ mm).

<u>Note S8. Theoretical analysis of the microlayer area factor η</u>

In our model, the microlayer area scaling factor η is used to capture the effect of the heat flux on the microlayer area fraction. The microlayer area is influenced by the nucleation density, so η can be determined based on the bubble nucleation density over the heated area. Here, we theoretically analyze the scaling factor η using a plain flat surface. At low heat flux, only a few bubbles nucleate on the surface, resulting in a small microlayer area fraction. As the heat flux increases, more bubbles nucleate and grow on the heated surface, leading to an increase in the microlayer area fraction. This area fraction can be estimated as: $A_{ml}/A_{total} = \sum_{i=1}^{N} A_{ml,i}/ A_{total} = NA_{ml,s}/A_{total}$, where N is the number of the nucleation sites, $A_{ml,i}$ is the microlayer evaporative area, A_{total} of the total heated area, and $A_{ml,s}$ is the averaged microlayer area of a single bubble. Since the bubble nucleation density n can be calculated as $n = N/A_{total}$, A_{ml}/A_{total} can then be rewritten as $A_{ml}/A_{total} = nA_{ml,s}$. Considering the assumption of $A_{ml}/A_{total} = \eta q_t''$, we can then obtain η as:

$$\eta = \frac{nA_{ml,s}}{q_t''}. \tag{S29}$$

Due to the lack of studies on the bubble nucleation density in liquid film boiling, we conducted a theoretical analysis of η based on our understanding of pool boiling over the uniform flat surface. For a uniform flat surface in pool boiling, CHF is approximately 110 W cm^{-2} at the saturation condition of water under one atmospheric pressure [25]. The maximum nucleation density $n_{\max}$ of a plain surface is approximately $0.25/(\pi D_b^2/4)$ [26]. Assuming the average diameter of the microlayer region is the bubble departure diameter ($D_{ml,s} = D_b$) [27], we can then obtain:

$$\eta_{pool} \approx \frac{\dfrac{0.25}{\pi D_b^2/4}\dfrac{\pi D_b^2}{4}}{q_{CHF}''} \approx 2.3\times10^{-3}\ \text{cm}^2\ \text{W}^{-1}. \tag{S30}$$

In the main text, we show that the value of $\eta = 2.15\times10^{-3}$ cm^2 W^{-1} is obtained by fitting the model prediction to our experimental data of liquid film boiling. This experimentally fitted value for liquid film boiling is close to the value of η on pool boiling as we derived above, indicating the correctness. However, it is important to note that η_{pool} from Eq. (S30) is obtained under the CHF condition for pool boiling on a plain surface. In our model, the value of η of liquid film boiling is needed to predict the HTC of liquid film boiling over a wide range of heat flux. Due to

the differences between these two conditions, even though the values are close, for the rigorousness we have not incorporated the theoretical solution η_{pool} into our model. We just use the value of η = 2.15×10^{-3} cm^2 W^{-1} obtained from the experimental fitting in our work.

Note S9. Sample fabrication, wetting characterization and liquid wicking experiments

In this work, we use thermal diffusion bonding method to fabricate the micromesh wicking structures with commercial copper meshes. Details about the fabrication process can be seen in our previous work [22]. We note that the fabricated micromesh surfaces here are all staggered-aligned, as shown in Figs. S7A-C. Samples s1, s2, and s7 have similar thickness but different spacing width, which are fabricated using the micromesh with different mesh sizes. To obtain micromesh samples with different porosity but the same thickness and spacing width (samples s3, s4, and s7), we use different compressing pressure to compress the multi-layer mesh with varying layer numbers to a similar thickness (Table S5). Instead, we use the same compressing pressure for multi-layer mesh with different layer numbers to fabricate micromesh samples (samples s5, s6, and s7) with different thickness but similar porosity and spacing width.

A goniometer (Krüss, DSA30S) is employed to measure the contact angle of water on a plain copper substrate. The measurement details have been explained in detail in our previous work [22]. Our results reveal the plain copper in our experiment has static contact angle of $\theta_S = 58.5 \pm 0.7°$, advancing contact angle of $\theta_A = 74.5 \pm 3.0°$, receding contact angle of $\theta_R = 8.4 \pm 0.8°$, and contact angle hysteresis $\Delta\theta = 66.1 \pm 3.8°$.

The wicking capability of the sample is tested to evaluate the absolute permeability. As shown in Fig. S7D, once the bottom of the sample is in contact with the water, the front line of the liquid is recorded by the high-speed camera (Photron FASTCAM SA-X2) at a frame rate of 500 fps to quantitatively calculate the wicking height h_w (the distance between the front line of the wicking liquid and the bulk water level) as a function of the square of time t. The relationship between the t and h_w can be determined by [28]

$$t = -\frac{h_w}{b} - \frac{a}{b^2}\ln\left(1 - \frac{b}{a}h_w\right), \tag{S31}$$

where $a = P_c K_w / \mu_l \varepsilon_w$ and $b = \rho_l g K_w / \mu_l \varepsilon_w$. Table S5 summarizes the structure properties of the tested samples.

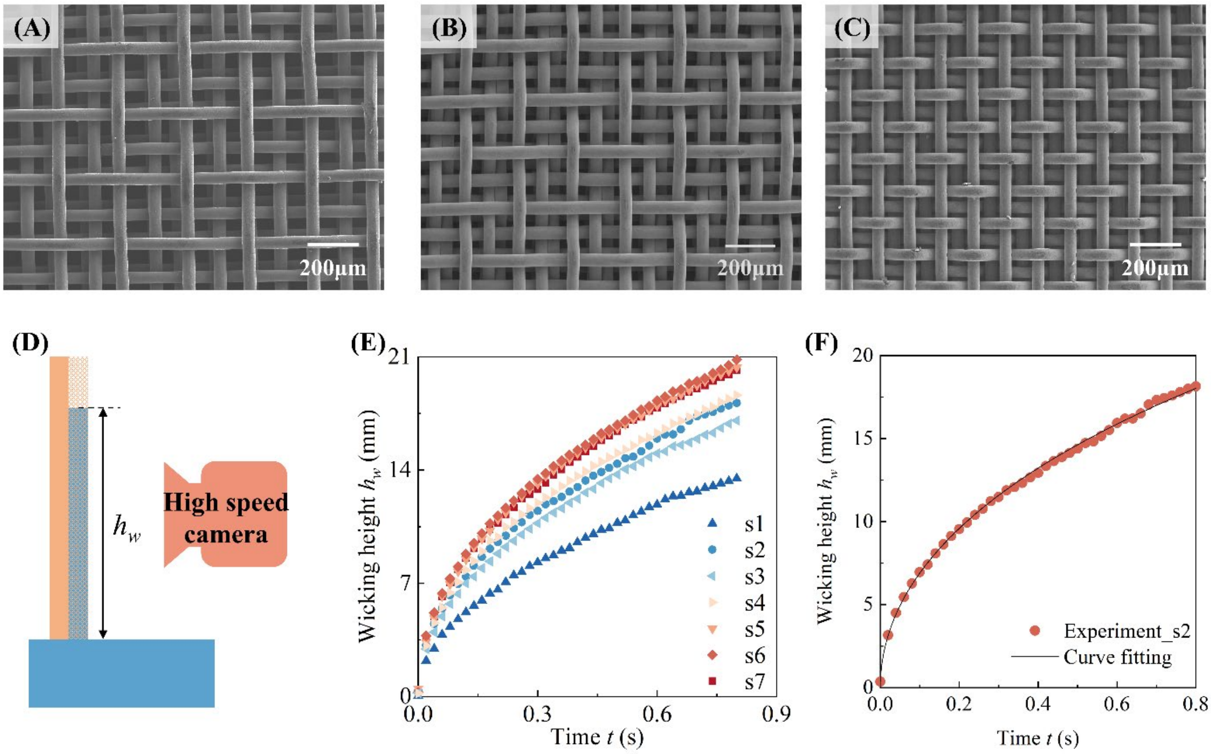

Figure S7. Scanning electron microscopy (SEM) images of micromesh structures with (A) s_w = 205μm, (B) s_w = 160 μm and (C) s_w = 77μm. (D) Schematic of the experimental setup for liquid wicking. (E) Water wicking distance h_w as a function of time t for all samples. (F) Experimental wicking distance of sample 2, along with its corresponding fitting curve by Eq. (S31).

Table S5. Structure properties of the tested micromesh surfaces

Sample no.	Wire diameter d_w (μm)	Spacing width s_w (μm)	Layer number n	Thickness δ_w (μm)	Porosity ε_w	Permeability K_w (10^{-10} m^2)
s1	47	77	4	268	57.4%	0.77
s2	50	160	4	264	70.8%	3.27
s3	50	205	6	261	63.9%	1.97
s4	50	205	5	267	71.3%	3.63
s5	50	205	5	313	74.9%	5.48
s6	50	205	6	377	75.0%	5.66
s7	50	205	4	260	75.9%	5.02

Note S10. Experimental setup, uncertainty analysis and repeatability for liquid film boiling

The experimental setup and the procedure of liquid film boiling experiments have been explained in detail in our previous work [1, 22] and are briefly summarized here. The experiments are conducted using a custom-built experimental system as shown in Fig. S8A. Before the experiment, DI water in the chamber is degassed by vigorously boiling for 2 h to purge non-condensable gas. The top of the tested samples is fixed at a height of 20 mm from the water level to maintain the 10×10 mm^2 heat input area above the water. A transparent window (100 mm in diameter) is installed, facing the front side of the tested surfaces for the visualization using the high-speed camera (Photron FASTCAM SA-X2). Before the experiment, the tested samples are well attached to the copper block using a solder sheet (Sn63Pb37). To eliminate the thickness changes of solder joints during the melting-solidification process, we measured the thickness of the solder joint for each sample. Cartridge heaters inserted at the base of the heating block can provide heated power to the sample. During the experiment, the entire copper block is insulated with polyether ether ketone (PEEK, k = 0.25 W $m^{-1}K^{-1}$) and glass fiber (k = 0.1 W $m^{-1}K^{-1}$) on all sides. Three K-type thermocouples (Omega) are inserted into the copper column to measure the temperature distribution of the copper square column and then used to calculate the surface temperature. During the experiment, each heat flux is maintained for 10-20 min until the steady state is reached, where the temperature changes at a rate of less than 0.2 K over a period of 3 min. As the heat power increases, data are recorded using a data acquisition system (Keithley DAQ6510) until the CHF.

As shown in Fig. S8B, the total heat flux q_t'' supplied to the sample can be calculated from,

$$q_t'' = -k_{Cu}\frac{dT_{Cu}}{dx}, \tag{S32}$$

where k_{Cu} is the thermal conductivity of the copper. The temperature gradient along the copper square column dT_{Cu}/dx is calculated using Taylor's backward series approximation (Fig. S6B),

$$\frac{dT_{Cu}}{dx} = \frac{3T_1 - 4T_2 + T_3}{2\Delta x_1}, \tag{S33}$$

where Δx_1 is the distance between the neighboring thermocouples in the heating block. The wall temperature (T_{wall}) of the tested surface can then be calculated as

$$T_{\text{wall}} = T_1 - q_t''\left(\frac{\Delta x_2}{k_{\text{Cu}}} + \frac{\delta_{\text{solder}}}{k_{\text{solder}}}\right), \tag{S34}$$

where δ_{solder} and k_{solder} are the thickness and the thermal conductivity of the solder joint, respectively. Δx_2 is the distance between the measurement position of the first thermocouple to the top surface of the copper block. The surface superheat ΔT is defined as the temperature difference between the wall temperature (T_{wall}) of tested samples and the saturated water vapor temperature (T_v)

$$\Delta T = T_1 - q_t''\left(\frac{\Delta x_2}{k_{\text{Cu}}} + \frac{\delta_{\text{solder}}}{k_{\text{solder}}}\right) - T_v. \tag{S35}$$

The total heat transfer coefficient h_t is calculated as

$$h_t = \frac{q_t''}{\Delta T}. \tag{S36}$$

Based on the error propagation, the uncertainty of heat flux can be determined by

$$\sigma(q_t'') = \sqrt{\left[\frac{\partial q_t''}{\partial k_{\text{Cu}}}\sigma(k_{\text{Cu}})\right]^2 + \left[\frac{\partial q_t''}{\partial T_i}\sigma(T_i)\right]^2 + \left[\frac{\partial q_t''}{\partial \Delta x_1}\sigma(\Delta x_1)\right]^2}. \tag{S37}$$

where the uncertainty of the thermal conductivity of copper $\sigma(k_{\text{Cu}})$ due to the change of temperature in experiments is ± 1% k_{Cu}, the uncertainty of the thermocouple $\sigma(T_i)$ is ± 0.1K, the uncertainty of the distance measurements $\sigma(\Delta x_1)$ is ± 0.1 mm. The uncertainty of wall temperature of the tested surface $\sigma(T_{\text{wall}})$ can be determined by

$$\sigma(T_{\text{wall}}) = \sqrt{\begin{gathered}\left[\frac{\partial T_{\text{wall}}}{\partial T_1}\sigma(T_1)\right]^2 + \left[\frac{\partial T_{\text{wall}}}{\partial q_t''}\sigma(q_t'')\right]^2 + \left[\frac{\partial T_{\text{wall}}}{\partial \Delta x_2}\sigma(\Delta x_2)\right]^2 \\ + \left[\frac{\partial T_{\text{wall}}}{\partial k_{\text{Cu}}}\sigma(k_{Cu})\right]^2 + \left[\frac{\partial T_{\text{wall}}}{\partial \delta_{\text{solder}}}\sigma(\delta_{\text{solder}})\right]^2 + \left[\frac{\partial T_{\text{wall}}}{\partial k_{\text{solder}}}\sigma(k_{\text{solder}})\right]^2\end{gathered}}, \tag{S38}$$

where the uncertainties of the distance measurements $\sigma(\Delta x_2)$ and the solder joint thickness $\sigma(\delta_{\text{solder}})$ are ± 0.1 mm and ± 0.025mm, respectively. The uncertainty of the thermal conductivity of solder joint $\sigma(k_{\text{solder}})$ is assumed to be the same with copper. The uncertainty of the superheat $\sigma(\Delta T)$ can be determined by

$$\sigma(\Delta T) = \sqrt{\left[\frac{\partial \Delta T}{\partial T_{\text{wall}}}\sigma(T_{\text{wall}})\right]^2 + \left[\frac{\partial \Delta T}{\partial T_v}\sigma(T_v)\right]^2}. \tag{S39}$$

Subsequently, the uncertainty of the heat transfer coefficient $\sigma(h_t)$ can be determined by

$$\sigma(h_t) = \sqrt{\left[\frac{\partial h_t}{\partial q_t''}\sigma(q_t'')\right]^2 + \left[\frac{\partial h_t}{\partial \Delta T}\sigma(\Delta T)\right]^2}. \tag{S40}$$

Figure S8C shows the temperature distribution inside the copper column at various heat fluxes. The R-squared values are all greater than 0.99996, confirming the earlier assumption of a one-dimensional linear distribution of temperature. To test the repeatability of the liquid film boiling measurement, three tests were done on each sample. After each run reaches the CHF point, the sample is cooled down back to nearly saturated vapor temperature and goes through the next run. The boiling curves of the three tests for sample s4 are shown in Fig. S8D, the CHF shows good repeatability within ± 2% from run to run.

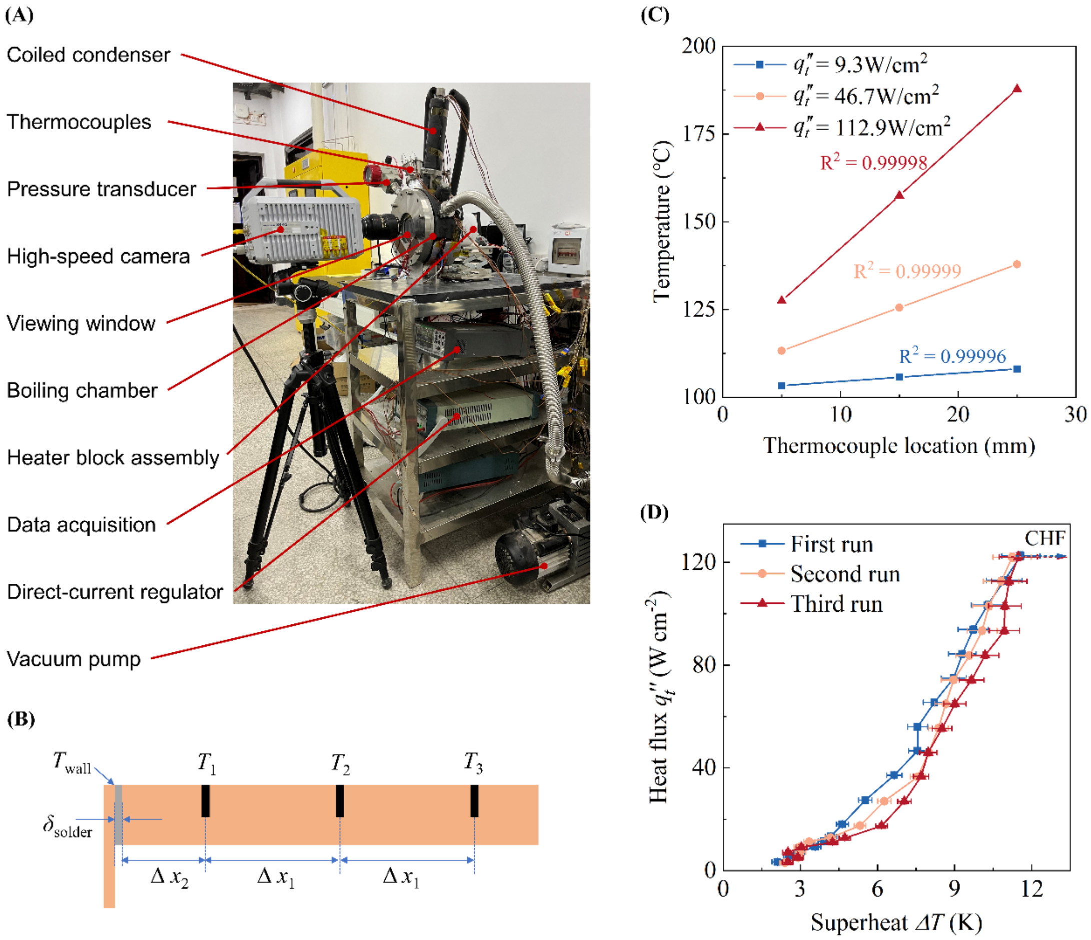

Figure S8. Experimental setup for capillary-driven liquid film boiling. (A) Photograph of the experimental setup. (B) Schematic of the copper heater block temperature measurement locations and nomenclature. (C) Measured temperature distribution of the copper column at different heat fluxes. (D) The repeatability of the boiling curve.

References

1. Wen R, Xu S, Lee Y-C *et al.* Capillary-driven liquid film boiling heat transfer on hybrid mesh wicking structures. *Nano Energy* 2018; **51**: 373-82.
2. Weibel JA, Garimella SV, North MT. Characterization of evaporation and boiling from sintered powder wicks fed by capillary action. *Int J Heat Mass Transf* 2010; **53**: 4204-15.
3. Cai SQ, Bhunia A. Geometrical effects of wick structures on the maximum phase change capability. *Int J Heat Mass Transf* 2014; **79**: 981-8.
4. Jeon S, Byon C. Effect of meniscus on the permeability of mono-layered and multi-layered packed spheres. *Appl Therm Eng* 2016; **107**: 1287-93.
5. Li C, Peterson GP. The effective thermal conductivity of wire screen. *Int J Heat Mass Transf* 2006; **49**: 4095-105.
6. Bongarala M, Hu H, Weibel JA *et al.* A figure of merit to characterize the efficacy of evaporation from porous microstructured surfaces. *Int J Heat Mass Transf* 2022; **182**: 121964.
7. Carman PC. Fluid flow through granular beds. *Chem Eng Res Des* 1997; **75**: S32-48.
8. Ngo I-L, Byon C. Permeability of microporous wicks with geometric inverse to sintered particles. *Int J Heat Mass Transf* 2016; **92**: 298-302.
9. Byon C, Kim SJ. The effect of meniscus on the permeability of micro-post arrays. *J Micromech Microeng* 2011; **21**: 115011.
10. Imura H, Kozai H, Ikeda Y. The effective pore radius of screen wicks. *Heat Transfer Eng* 1994; **15**: 24-32.
11. Adera S, Antao D, Raj R *et al.* Design of micropillar wicks for thin-film evaporation. *Int J Heat Mass Transf* 2016; **101**: 280-94.
12. Peterson GP, Fletcher LS. Effective thermal conductivity of sintered heat pipe wicks. *J Thermophys Heat Transfer* 1987; **1**: 343-7.
13. Ranjan R, Murthy JY, Garimella SV. Analysis of the wicking and thin-film evaporation characteristics of microstructures. *J Heat Transf* 2009; **131**: 101001.
14. Zhang C, Palko JW, Barako MT *et al.* Design and optimization of well-ordered microporous copper structure for high heat flux cooling applications. *Int J Heat Mass Transf* 2021; **173**: 121241.
15. Brinkman HC. A calculation of the viscous force exerted by a flowing fluid on a dense swarm of particles. *Appl Sci Res* 1949; **1**: 27.
16. Kaviany M. *Principles of Heat Transfer in Porous Media*. New York: Springer, 2012.
17. Wang CY. A fixed-grid numerical algorithm for two-phase flow and heat transfer in porous media. *Numerical Heat Transfer, Part B: Fundamentals* 1997; **32**: 85-105.
18. Dai X, Yang F, Yang RG *et al.* Micromembrane-enhanced capillary evaporation. *Int J Heat Mass Transf* 2013; **64**: 1101-8.
19. Sudhakar S, Weibel JA, Garimella SV. A semi-empirical model for thermal resistance and dryout during boiling in thin porous evaporators fed by capillary action. *Int J Heat Mass Transf* 2021; **181**: 121887.
20. Lu Z, Preston DJ, Antao DS *et al.* Coexistence of pinning and moving on a contact line. *Langmuir* 2017; **33**: 8970-5.
21. Alhosani MH, Li H, Alketbi AS *et al.* Enhanced liquid propagation and wicking along nanostructured porous surfaces. *Adv Eng Mater* 2021; **23**: 2100118.
22. Tu Y, Zhou J, Lin S *et al.* Plausible photomolecular effect leading to water evaporation

exceeding the thermal limit. *Proceedings of the National Academy of Sciences* 2023; **120**: e2312751120.
23. Schrage RW. *A Theoretical Study of Interphase Mass Transfer*. New York: Columbia University Press, 1953.
24. Carey VP. *Liquid-vapor Phase-change Phenomena: An Introduction to the Thermophysics of Vaporization and Condensation Processes in Heat Transfer Equipment*. New York: CRC Press, 2018.
25. Zuber N. *Hydrodynamic aspects of boiling heat transfer*: United States Atomic Energy Commission, Technical Information Service, 1959.
26. Zhang L, Gong S, Lu Z *et al.* Boiling crisis due to bubble interactions. *Int J Heat Mass Transf* 2022; **182**: 121904.
27. Chen Z, Haginiwa A, Utaka Y. Detailed structure of microlayer in nucleate pool boiling for water measured by laser interferometric method. *Int J Heat Mass Transf* 2017; **108**: 1285-91.
28. Fries N, Dreyer M. An analytic solution of capillary rise restrained by gravity. *J Colloid Interface Sci* 2008; **320**: 259-63.
